# PRIDE Inspector Toolsuite: Moving Toward a Universal Visualization Tool for Proteomics Data Standard Formats and Quality Assessment of ProteomeXchange Datasets* [Fn FN2]

**DOI:** 10.1074/mcp.O115.050229

**Published:** 2015-11-06

**Authors:** Yasset Perez-Riverol, Qing-Wei Xu, Rui Wang, Julian Uszkoreit, Johannes Griss, Aniel Sanchez, Florian Reisinger, Attila Csordas, Tobias Ternent, Noemi del-Toro, Jose A. Dianes, Martin Eisenacher, Henning Hermjakob, Juan Antonio Vizcaíno

**Affiliations:** From the ‡European Molecular Biology Laboratory, European Bioinformatics Institute (EMBL-EBI), Wellcome Trust Genome Campus, Hinxton, Cambridge, CB10 1SD, UK;; §Ruhr-Universität Bochum, Medizinisches Proteom-Zenter, Medical Bioinformatics, ZKF, E.142, Universitätsstr. 150, D-44801 Bochum, Germany;; ¶Division of Immunology, Allergy and Infectious Diseases, Department of Dermatology, Medical University of Vienna, Austria;; ‖Department of Proteomics, Center for Genetic Engineering and Biotechnology, Ciudad de la Habana, Cuba

## Abstract

The original PRIDE Inspector tool was developed as an open source standalone tool to enable the visualization and validation of mass-spectrometry (MS)-based proteomics data before data submission or already publicly available in the Proteomics Identifications (PRIDE) database. The initial implementation of the tool focused on visualizing PRIDE data by supporting the PRIDE XML format and a direct access to private (password protected) and public experiments in PRIDE.

The ProteomeXchange (PX) Consortium has been set up to enable a better integration of existing public proteomics repositories, maximizing its benefit to the scientific community through the implementation of standard submission and dissemination pipelines. Within the Consortium, PRIDE is focused on supporting submissions of tandem MS data. The increasing use and popularity of the new Proteomics Standards Initiative (PSI) data standards such as mzIdentML and mzTab, and the diversity of workflows supported by the PX resources, prompted us to design and implement a new suite of algorithms and libraries that would build upon the success of the original PRIDE Inspector and would enable users to visualize and validate PX “complete” submissions. The PRIDE Inspector Toolsuite supports the handling and visualization of different experimental output files, ranging from spectra (mzML, mzXML, and the most popular peak lists formats) and peptide and protein identification results (mzIdentML, PRIDE XML, mzTab) to quantification data (mzTab, PRIDE XML), using a modular and extensible set of open-source, cross-platform libraries. We believe that the PRIDE Inspector Toolsuite represents a milestone in the visualization and quality assessment of proteomics data. It is freely available at http://github.com/PRIDE-Toolsuite/.

The amount of publicly available mass spectrometry (MS)-based proteomics data is rapidly increasing in quality and quantity. This is due to the guidelines promoted by several scientific journals like *Molecular and Cellular Proteomics* (MCP) and by funding agencies (1). Additionally, there is a growing perception in the community that sharing data is a good scientific practice and beneficial for the field (2). The ProteomeXchange (PX) Consortium was formally started in 2011 to overcome the challenges in MS proteomics data sharing and dissemination (3, 4) by implementing standard pipelines and promoting collaboration, developing an international consortium of major stakeholders in the domain. At present, it includes the PRoteomics IDEntifications (PRIDE) database (5), PeptideAtlas (6) and the related resource PeptideAtlas SRM Experiment Library (PASSEL) (7) and the Mass Spectrometry Interactive Virtual Environment (MassIVE, http://massive.ucsd.edu/).

In parallel with the PX Consortium, different community open standard formats have been developed over the last few years, under the auspices of the Proteomics Standards Initiative (PSI). In the context of bottom-up MS/MS approaches, the most adopted XML-based standards are: mzML (8) to store the “primary” MS data (the mass spectra and chromatograms) and mzIdentML (9) to report peptide identifications as well as the inferred protein identifications, including posttranslational modifications. The mzIdentML format, among other supported features, can represent protein-inference-related information using protein ambiguity groups, provides detailed ranking of peptide spectrum matches (PSMs) and can store rich metadata about the search parameters used in the analysis.

In addition, recently, the tab-delimited format mzTab (10) was developed to represent both identification and quantification results in the same file, enabling the reporting of the experimental results at different levels of detail. It is important to note that, while processed results are stored, the corresponding mass spectra are not stored in the mzIdentML and mzTab files. However, this information can be linked to mass spectra data available in external file formats (including the data standard mzML).

The increasing adoption of standard data formats (11) facilitates the validation, reproducibility, and comparability of results produced by different instruments and software platforms. In addition, efforts can be concentrated in the development of visualization and analysis tools supporting the data standards, rather than the wide variety of data formats available in the field.

PX resources heavily rely on open data standard formats. At present, there are two different PX submission modes: “complete” and “partial.” In both types, processed identification results are mandated for each data submission. The difference lies within the file formats in which these processed results are provided. A complete submission implies that, after the files have been submitted, it is possible for the receiving repository (*e.g.* PRIDE, MassIVE^1^) to connect the processed results directly with the mass spectra, enabling visualization and quality assessment. For the repositories, this requirement can be achieved if the processed identification results are available in open file formats (*e.g.* mzIdentML, mzTab, or the older PRIDE XML, the original PRIDE data format) and the mass spectra files are included in the submission (12). In the case of partial submissions, processed identification results are accessible in the different nonstandard file formats output by each software and/or analysis pipeline. As a result, the files are available for download but the visualization of the data is often not possible without access to the original analysis software used. There are a few standalone tools for the visualization of MS proteomics data (13). Among these that are open source or free-to-use (14), it is worth highlighting Scaffold Viewer (15), Thermo MSF Viewer, ProteoIDViewer (16), TOPPView (17), and MS-Viewer (18). Overall, their main limitation is that these tools are mostly focused on one single format or on nonstandard data formats.

The original PRIDE Inspector tool (19) was developed as an open source standalone tool to enable the visualization and validation of proteomics data in PRIDE. The main motivation behind the project was to develop a user-friendly visualization tool for researchers to be able to interact with and take advantage of the growing data available in PRIDE. The initial implementation focused on visualizing PRIDE data (*via* the PRIDE XML format), although mzML was also supported. PRIDE Inspector has become the *de facto* visualization tool for PRIDE data for many researchers since at present the PRIDE web interface supports a subset of its functionality. However, the original PRIDE Inspector tool had some limitations in terms of software architecture and supported formats and it lacked some functionality for quality assessment and for quantitative data. To overcome these limitations, we decided to develop a new set of algorithms, libraries, and tools for the PRIDE Inspector Toolsuite, suitable to the evolving needs of the field. We then extended the original scope of the PRIDE Inspector tool by supporting the new PSI standard formats mzIdentML and mzTab and the wide variety of mass spectra file formats used currently. In addition, new functionalities were developed for improving the data visualization, validation, and quality assessment.

In this manuscript we describe the PRIDE Inspector Toolsuite, including its new features and supported data formats. PRIDE Inspector Toolsuite represents a feasible way to visualize annotated spectra coming from a wide variety of tools, as mandated by MCP (http://www.mcponline.org/site/misc/annotated_spectra.xhtml). We are certain that researchers and in particular data submitters or researchers interested in publicly available data at ProteomeXchange resources will greatly benefit from it. It is freely available to download at http://github.com/PRIDE-Toolsuite.

## MATERIALS AND METHODS

### 

#### 

##### Design and Implementation

The PRIDE Inspector Toolsuite is written in Java, ensuring that can be used in different operating systems such as Microsoft Windows, Mac OS, and Linux. The Toolsuite is divided in two main groups of libraries: (i) PRIDE-Utilities (https://github.com/PRIDE-Utilities), which contains the set of algorithms and libraries for data handling, validation, and quality assessment, and (ii) PRIDE-Toolsuite (https://github.com/PRIDE-Toolsuite), containing the set of graphical user interface components and tools (Table I). The code is distributed as open source under the very permissive Apache License, version 2.0. Supplemental File S1 is provided as an extensive PRIDE Inspector Toolsuite guide for users.

**Table I:** Organization of the PRIDE Inspector Toolsuite modules

	Library or GUI component	Description	GitHub repository
PRIDE-Utilities	PRIDE Utilities	It contains functionalities shared by different PRIDE Toolsuite libraries such as controlled vocabulary data structures and prediction algorithms of peptide/protein properties	https://github.com/PRIDE-Utilities/pride-utilities
	PRIDE Data Object Model (*ms-data-core-api*)	Data model representation of MS proteomics data with special emphasis in metadata information	https://github.com/PRIDE-Utilities/ms-data-core-api
	PRIDE Protein Inference	It implements a set of protein inference algorithms, coupled with the *ms-data-core-api* data model	https://github.com/PRIDE-Utilities/pride-protein-inference
	PRIDE Modification	It retrieves the information from the main protein modification controlled vocabularies and/or ontologies (Unimod and PSI-MOD)	https://github.com/PRIDE-Utilities/pride-mod
PRIDE-Toolsuite	PRIDE Inspector Quality Chart	Chart library developed using Java Swing and JFreeChart, which provides a way to assess the quality of MS experiments	https://github.com/PRIDE-Toolsuite/inspector-quality-chart
	PRIDE Spectrum Browser	Java Swing library to visualize and annotate MS/MS spectra, chromatograms and fragment annotations	https://github.com/PRIDE-Toolsuite/inspector-mzgraph-browser
	PRIDE Inspector Tool	Desktop application tool	https://github.com/PRIDE-Toolsuite/pride-inspector
	PRIDE Inspector Toolsuite examples	Set of example files from different sources (mzIdentML, PRIDE XML, mzTab, and mass spectra files) that can be used for testing purposes	https://github.com/PRIDE-Toolsuite/inspector-example-files

The development of PRIDE Inspector Toolsuite had several main goals

• Provide support for the major PSI standard formats that can be used at present for performing PX complete submissions;

• Provide support for all the experimental information available in an average proteomics experiment, ranging from spectra and peptide/protein identifications to quantification results;

• Reuse existing application programming interfaces and code libraries;

• Enable the reuse of each library in other proteomics packages such as PX-related submission and annotation pipelines and other third-party tools;

• Each tool should be accessible through a graphical user interface to provide a rich and user-friendly experience to the functionality provided; and

• Improve the original PRIDE Inspector tool by supporting the new use cases required by PRIDE users and the community as a whole.

To achieve these goals, the development of the PRIDE Inspector Toolsuite was based on modular programming techniques in which all the functionalities of a program are separated into independent, interchangeable modules. Each of them contains everything necessary to execute only one aspect of the desired functionality with minimum resource overhead. As such, the PRIDE Inspector Toolsuite consists of eight different libraries and graphical user interface components (Table I): PRIDE Utilities (*pride-utilities*), PRIDE Data Object Model (*ms-data-core-api*) (20), PRIDE Protein Inference (*pride-protein-inference*) (21), PRIDE Modification (*pride-mod*), PRIDE Inspector Quality Chart (*inspector-quality-chart*), PRIDE Spectrum Browser (*inspector-mzgraph-browser*), PRIDE Inspector tool (*pride-inspector*), and PRIDE Inspector Toolsuite examples (*inspector-example-files*).

##### MS/MS Spectrum Automatic Fragmentation Annotation Algorithm

**Fig. 1.**
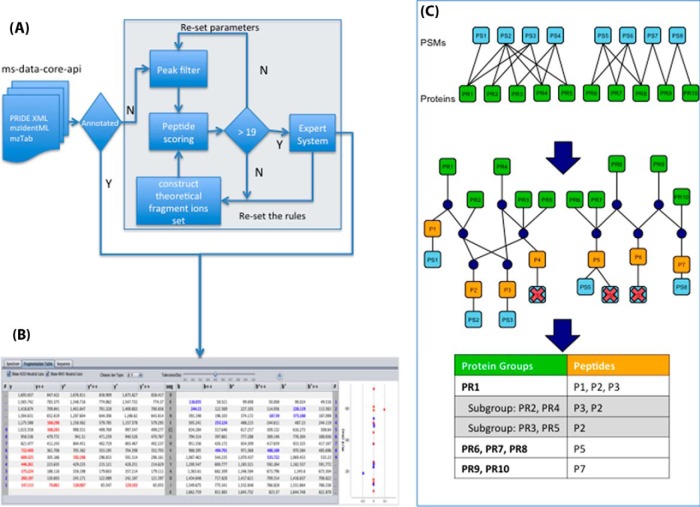
(*A*) Workflow explaining the MS/MS ion annotation algorithm. (*B*) Screenshot of the MS/MS ion annotation table, highlighting the assigned ions. It also shows the “delta mass” of each fragment ion. (*C*) Schema of the protein inference workflow. The top part of the figure shows an example illustrating the relationship between PSMs and proteins. The middle part represents the structure used internally, which contains nodes for PSMs (in light blue), peptides (in orange), proteins (in green), and also the necessary nodes (dark blue) to maintain the structure. For more details, see the main text.

In many cases, mass spectrum fragmentation information is not available in the standard file formats. Generally, it is not mandatory to provide this information in the supported files (*e.g.* mzIdentML and/or PRIDE XML), and therefore exporters/converters often do not take this information into account when generating these files. In other cases, the provision of this information is not supported at present (*e.g.* mzTab), or it can also happen that even the original search engine output files do not contain this information (*e.g.* Sequest .out files), so it cannot be exported. Therefore, a fragment ion annotation algorithm was developed to facilitate the interpretation of MS/MS spectra (Fig. 1*A*). It is important to note that PRIDE data are highly heterogeneous (different instruments, analytical methods, precision settings, etc). Therefore, it is challenging to provide an annotation system that can fit all possible use cases.

Briefly, the tandem mass spectrum peak list is first separated into windows of 100 *m/z* units. In each window, the top *i* intensity peaks are chosen, where *i* represents the peak depth. The cumulative binomial probability previously used in other algorithms (22, 23) is given to the generated fragment ion annotations, representing the probability of randomly matching at least the given number of fragment ions to the tandem mass spectrum, which is calculated by using the total number of fragment ions for the given peptide (*N*), the number of ions matched to the spectrum (*n*), and the probability of matching a peak (*p*). This probability can be used as a simple filter to block the generation of the fragment ion annotation for aberrant identifications or peptides with incorrect metadata annotations (*e.g.* wrong modification, charge, or mass). For matching the fragment peaks, a simple expert system is used (Supplemental File S1, Section 6). The filtered peaks are then annotated, highlighting the corresponding fragment ions in the mass spectrum viewer (Fig. 1*B*). The algorithm is available in the PRIDE Spectrum Browser library (http://github.com/PRIDE-Toolsuite/inspector-mzgraph-browser) (Supplemental File S1, Section 6). It is important to highlight that this algorithm only runs when the information is not available in the original files.

##### Protein Inference Algorithms

The PRIDE Protein Inference (*pride-protein-inference*) module is based in our toolbox for Protein Inference (called PIA) (21) and includes different algorithms for performing protein inference analysis. Currently, the module provides two protein inference methods: (i) “Report all,” which is the simplest inference method, just returning any possible protein group after the combination of all the search results and (ii) “Occam's razor,” which uses the principle of parsimony to report a minimal set of proteins that can explain the occurrence of all the identified peptides. To achieve this (Fig. 1*C*), in a cluster consisting of peptides and associated proteins, the protein groups are reported ordered by the number of subsumed peptides, starting with the groups containing the highest number of peptides, until all the peptides are assigned to a given group. Protein groups, which are completely subsumed by another group, are reported as subgroups. Figure 1*C* illustrates an example. There are two clusters (*i.e.* parts of the data that are independent for the determination of the protein inference): PSMs PS1-PS4 for the proteins PR1-PR5 and PSMs PS5-PS8 for the proteins PR6-PR10. All supported input formats contain the PSM to protein mappings, which are used to build a tree-like intermediate graph, containing the correspondence from PSMs to peptides to proteins (Fig. 1*C*, *middle*) in a fast and accessible way (20). The graph introduces special nodes (depicted in blue), which are necessary to maintain the structure. It is then easy to get all proteins connected to one PSM while moving upward in the graph or to get all PSMs of a protein moving in the other direction. Due to the filtering using a given score threshold, in the example, the PSMs PS4, PS6, and PS7 are removed, as highlighted in the intermediate graph. This filtering finally leads to the final reporting of three protein groups, of which the group with protein PR1 also contains two subgroups (see table in Fig. 1*C*). The current implementation supports three main scoring alternatives for each protein: (i) the “multiplicative scoring,” which multiplies the scores of the contributing PSMs; (ii) the “geometric mean scoring,” which calculates the *n*-th root of the product of *n* contributing PSMs; and (iii) the “additive scoring,” which simply adds up all the contributing PSM scores.

If a given file (*e.g.* mzIdentML, mzTab) does not contain any protein information (it only contains PSMs), these algorithms can be used to perform the protein inference analysis. This is especially useful for the output of search engines that do not perform any protein inference analysis themselves (*e.g.* MS-GF+, X!Tandem).

##### Metadata Components and Libraries

The PRIDE Inspector Toolsuite contains many additional features intended to facilitate the handling of MS proteomics data. The PSI data standard formats include rich metadata information, which is normally provided using controlled vocabulary or ontology terms, including, but not limited to, details on contacts, experimental protocols, instrumentation, software processing, journal references, search database annotations, protein sequences, and protein modifications (including posttranslational modifications). The PRIDE Utilities (http://github.com/PRIDE-Utilities/pride-utilities) and PRIDE Modifications libraries (http://github.com/PRIDE-Utilities/pride-mod) can automatically map controlled vocabulary annotations between different controlled vocabularies/ontologies such as PSI-MS (24), PSI-MOD (25), Unimod (26), and the PRIDE Controlled vocabulary (27). These modules can then homogenize all the terms and concepts included in the annotations, making this process invisible to the users.

## RESULTS

**Fig. 2.**
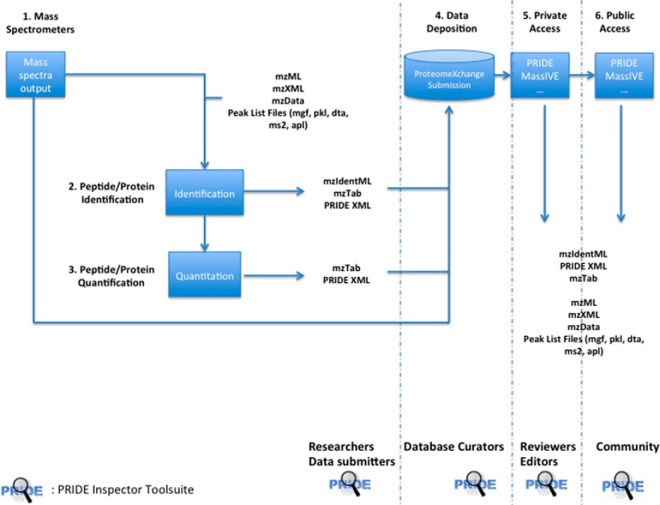
**PRIDE Inspector Toolsuite can be used in every stage of a proteomics data workflow based on standard file formats;** (1) generation of mass spectrometers output files; (2) peptide/protein identification step; (3) peptide/protein quantification step; (4) data deposition in any of the ProteomeXchange resources (PRIDE, MassIVE); (5) private access. Journal reviewers and editors can access the submitted files (password protected); and (6) public access. The data submission is made publicly available after the acceptance of the manuscript.

The PRIDE Inspector Toolsuite, consisting of eight different libraries (see the Methods section), can be used at different levels of the MS proteomics data analysis pipeline to assist data analysis, visualization, and quality assessment of the originally generated data, before data submission to a public repository (usually linked to the manuscript review process) (5), and also for studying datasets available in the public domain in PX resources (Fig. 2). We next describe the main features and functionality of the PRIDE Inspector tool, as the main software tool of the Toolsuite.

### 

#### 

##### Support for New File Formats

The updated PRIDE Inspector tool integrates all the libraries and algorithms of the Toolsuite, enabling the visualization, validation, and quality assessment of proteomics experiments. Any application built upon the PRIDE Data Object Model (*ms-data-core-api*) is largely format agnostic for some of the most popular formats in the field. PRIDE Inspector supports the handling and visualization of different experimental output files, ranging from mass spectra (mzML, mzXML, and the most popular peak lists formats), peptide, and protein identification results (mzIdentML, PRIDE XML, mzTab) to quantification data (mzTab, PRIDE XML).

Due to its increased adoption, a streamlined full support for the mzIdentML format is the first aspect that needs to be highlighted. For this purpose, mzIdentML files exported from a variety of sources, including many of the most popular proteomics analysis software, have been tested and are fully supported. This includes MS-GF+ (28), Mascot (*Matrix Science,* from version 2.4), ProteinPilot (*AB SCIEX*), PEAKS (29), Scaffold (15), MyriMatch (30), and PeptideShaker (31). In addition, it is also important to note that if the open source analysis tool PeptideShaker is used for the analysis, the output of additional open source search engines are fully supported *via* the PeptideShaker mzIdentML export functionality: X!Tandem (32), MS Amanda (23), OMSSA (33), Tide (34), Andromeda (35), and Comet (36).

Unlike PRIDE XML, mzIdentML, and mzTab files do not contain the mass spectra data. Instead they reference the mass spectra in linked external files. Jointly with the corresponding mzIdentML/mzTab files or independently as individual files, the most popular used mass spectra formats are fully supported: mzML and its predecessors, the XML-based formats, mzData and mzXML (37), and the highly used text-based formats, Mascot Generic Format#(MGF) DTA, Micromass PKL, MaxQuant apl (38), and MS2 (39). The exact formatting of these references depends on each particular file format. However, PRIDE Inspector hides this process from the end user, using the functionality provided by the jmzReader library (40). Example mzIdentML files (including the corresponding mass spectra files) coming from a variety of sources to test the functionality of the software are available at https://github.com/PRIDE-Toolsuite/inspector-example-files. As a key point, support for quantitative data has been greatly improved in the Toolsuite by reading and writing mzTab files. To our knowledge, the PRIDE Inspector Toolsuite is the first application that supports mzTab files containing identification and quantification results. Files from three different mzTab exporters have been tested and are fully supported: Mascot (from version 2.5), jmzTab-based data converters (https://github.com/PRIDE-Utilities/jmzTab), and IsoQuant (41) (Table II). Finally, support for the PRIDE XML format is maintained to ensure support for older data as well as existing converters and exporters.

**Table II:** List of supported file formats in PRIDE Inspector Toolsuite, including the list of the exporters explicitly tested and supported (by September 2015)

	File format	Software provider	New in PRIDE Inspector	Available panels in PRIDE Inspector	Used Application Programming Interface
Mass spectra file formats	mzML	ProteoWizard and others	No	Metadata	jmzML
	mzXML		Yes	Mass Spectrum	jmzReader
	mzData		Yes	Summary Charts	jmzReader
	Peak list files (mgf, ms2, dta, pkl, apl)		Yes	Mass Spectrum, Summary Charts	jmzReader
Identification file formats	PRIDE XML	PRIDE Converter 2	No		pride-jaxb
		MS-GF+			
		Mascot (version 2.4)			
		Scaffold			
		ProteinPilot		Metadata	
		Myrimatch		Protein	
		ProteoAnnotator		Peptide	
	mzIdentML	PeptideShaker (X!Tandem, MS Amanda, OMSSA, Tide, Andromeda and Comet)	Yes	Mass Spectrum (if mass spectra file is associated)	jmzIdentML
		PEAKS		Summary Charts	
		IsoQuant			
		Pep2pro			
	mzTab (version 1.0)	jmzTab converter		Metadata	jmzTab
		Mascot (version 2.5)	Yes	Protein	
		IsoQuant		Peptide	
				Mass Spectrum (if mass spectra file is associated)	
				Summary Charts	
Quantification file formats	mzTab (version 1.0)	Mascot (version 2.5)	Yes	Metadata	jmzTab
				Protein	
				Peptide	
	PRIDE XML	PRIDE Converter 2	No	Mass Spectrum (if peak list file is associated)	pride-jaxb
				Quantification	
				Summary Charts	

As mentioned earlier, this support of multiple file formats is facilitated by the Data Object Model layer in the Toolsuite (*ms-data-core-api*), which provides a unified access interface to MS data, independent of the underlying file format's specific details. This interface provides methods to access and retrieve information on experimental metadata, mass spectra, peptides, proteins, and protein modifications from the source files, including identification and quantification data (Fig. 2).

##### Description of the General Visualization Features

The PRIDE Inspector graphical user interface was redesigned to provide a lightweight and robust way to visualize, validate, and perform quality assessment of MS proteomics data (Supplemental File S1, Section 2). The tool can be browsed through its different panels and views, each focusing on a specific aspect of the data. The original version of the tool (19) has been completely redesigned to support new data types and concepts such as protein groups and automated MS/MS fragment ion annotations. In parallel, most of the features available in the original tool have been maintained and in many cases enhanced. It must be taken into account that, depending on the type of information available for a given file format (Table II), some views in the tool can remain inactive (Table II). Six main views of the data are supported: metadata (overview), proteins, peptides, spectra, quantification, and charts.

##### Visualization of the Experimental Metadata

The “Overview” tab includes a metadata panel with information about the searched database, peptide/protein identification protocols and software parameters (Supplemental Figs. 1–4). Within this tab, the “Experiment General” view shows an overview of the experiment, including the title, instrument, references and contacts (Supplemental Fig. 1). The “Sample Protocol” and “Instrument Processing” views show general metadata about the sample and the instrument used in the experiment, respectively. This information is present in PRIDE XML, mzTab, and the annotated mass spectra files such as mzML, mzXML, and mzData files (Supplemental Fig. 3). The “Identification Protocol” view includes the search database used for the analysis and the search parameters and thresholds (Supplemental Fig. 4). It can capture multiple identification protocols and present the metadata information for all of them.

##### Visualization of Protein and Peptide Information

**Fig. 3.**
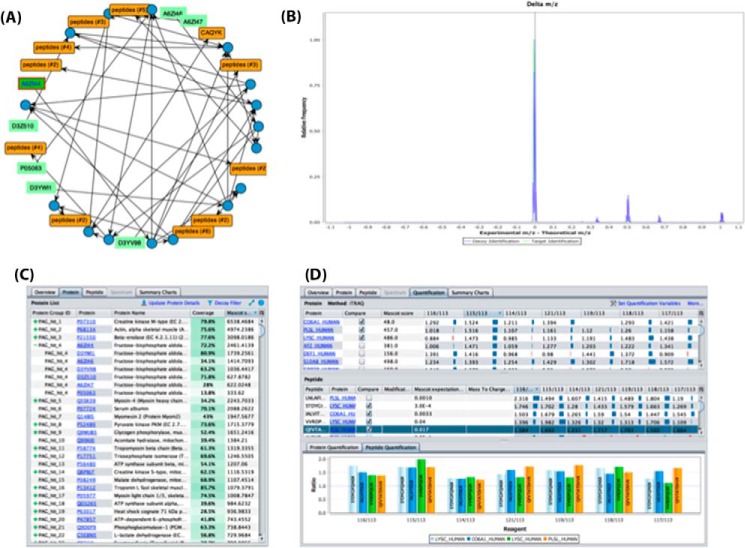
**Screenshots showing some of the novel graphical features of the PRIDE Inspector tool:** (*A*) visualization of protein groups; (*B*) chart of “delta *m/z* distribution” including target (in green) and decoy peptides (in blue); (*C*) “protein view” containing protein inference information—the + sign should be clicked to show the proteins contained in one protein group; and (*D*) “Quantification view,” which provides expression level details at the protein and peptide level across different samples.

The second tab (“Protein view”) is possibly the most interesting one for most of the users (Fig. 3*C*). For each identified protein, all the peptide identifications, protein modifications, and the corresponding mass spectra are displayed in a concise manner in the lower part of the panel (see next section for more details about the “Spectrum Browser”). Metadata related to the protein identifications (*e.g.* search engine or the searched database) are also provided there. The original protein sequences for each identification can be provided in the sequence panel if this information is included in the mzIdentML, PRIDE XML, or mzTab files (this information is optional). However, if this is not the case, users can still use the integrated access to existing web services to retrieve the protein sequences from the corresponding protein sequence databases (Supplemental File S1, Section 4), by clicking the “Update Protein Details” option. The protein table will show a heatmap (13) to represent the identified proteins and their sequence coverage (Fig. 3*C*). The “Protein sequence viewer” can then highlight different features such as identified peptides and modifications.

Protein identification information is optional in mzTab and mzIdentML, so in some cases, only PSM related data is available, depending on the exporter/converter that generated the files. In the original version of PRIDE Inspector the handling of protein inference was limited since protein groups were not supported in PRIDE XML. If protein inference information is not available, the Toolsuite now enables users to perform the protein inference analysis as explained in the Methods section, using by default the “Occam's razor” algorithm. If protein inference information is available, the protein groups are then used to represent the ambiguity of the peptide—protein assignments (Fig. 3*C*). A tree-like table is used to represent this structure where each root node of the table represents the anchor protein and the rest are the proteins belonging to the same group (Fig. 3*C*). A new visualization component was developed to show shared peptide evidences between different protein identifications. It has three different layouts: an intuitive tree-like protein layout, circle, and force-directed (Fig. 3*A*). As a key feature, the new component enables filtering PSMs by search engine scores in order to analyze the protein inference graph (Supplemental File S1, Section 5).

The third tab corresponds to the “Peptide view.” It focuses on the peptide identifications and the PSMs, including search engine scores and protein modifications. In addition, it can generate useful information not present in the original files such as the isoelectric point (42) (Supplemental Fig. 5). It also shows for all identified peptides the corresponding PSMs, including *e.g.* PSMs scores and modifications. For both the “Protein View” and “Peptide View,” the difference between experimental and theoretical mass-over-charge ratio (delta *m/z* value) is highlighted (Supplemental File S1, Section 3.1). In both views, it is also possible to filter out the PSMs using the rank (if this information is available in the file), the decoy identifications, and as such, to estimate the peptide false discovery rate.

##### Visualization of the Mass Spectrum Fragment Annotations

The mass spectrum component provides annotated mass spectra highlighting the identified sequences in both the “Protein View” and “Peptide View” panels (Supplemental Figs. 5 and 6). The annotations are either available in the original files or generated automatically, as explained in Methods. It should be noted that in mzTab files the fragment ion annotations are never present (there is not an established mechanism to do it in version 1.0 of mzTab), and for mzIdentML and PRIDE XML, this information is optional and often not present. One of the reasons is that the presence of this information considerably increases the file size, which is why this information is often omitted. If the fragment annotations are not provided in the files, the *inspector-mzgraph-browser* and *pride-utilities* libraries can generate automatic fragment annotations, as explained in the Methods section. The scores generated *via* the implemented fragment ion annotation algorithm do not replace the original PSM scores provided by the search engines. This is a key requirement for reviewers and editors, curators, and journals like MCP, which mandates in its guidelines that annotated MS/MS spectra are made available in some cases to support the publication of the corresponding manuscripts.

Users can also drag the mouse between two peaks in the spectrum to display the mass difference. This type of visualization is commonly used in other tools such as Mascot (43), PEAKS (29), and Scaffold (15). Therefore, users can now check the originally provided fragment ion annotations based on the information available in the mzIdentML or PRIDE XML files (Supplemental Fig. 6). In addition, the “Spectrum Browser” can summarize the sequence-derived fragment ions in a table (Supplemental Fig. 6 and Supplemental File S1, Section 6). A chart displays the mass difference between the calculated and experimental fragment ion mass values in the same units used to specify theerror mass tolerance. If the distribution of the delta mass difference is too high, this is an indication of a possible incorrect identification even if the search engine score is good (44).

The PRIDE Inspector “Mass Spectrum Browser” has been extensively used as a standalone mass spectrum viewer (40, 45). In addition to fragment ion annotation, this software component was then redesigned in order to support other new features such as precursor ion selection (Supplemental Figs. 8 and 9). It uses the *inspector-mzgraph-browser* module to access and visualize all mass spectra in the file, not only the identified ones (Supplemental Fig. 7). In addition, two more features requested by the users are now supported:

- Precursor annotation information—If the spectrum has more than one precursor ion associated, the users are able to see related information such as the charge state and precursor ion intensity.
- Column to highlight whether the spectrum is identified or unidentified, giving for instance the possibility to sort spectra by this column value and see the difference in total ion intensity or precursor ion charge, between identified and unidentified spectra in a given experiment. This option combined with the high number of spectra file formats supported provides a unique feature among other tools (18, 46). For example, for data reanalysis pipelines, it can be interesting to filter the unidentified spectra in a particular PX dataset that have a total ion count higher than a specific threshold.

Finally, chromatograms can also be visualized in this panel if available, in the case of mzML files (Supplemental Fig. 8).

##### Visualization of Quantification Information

The “Quantification view” panel is only active for PRIDE XML and mzTab files containing quantification information (Fig. 3*D*). This panel has been completely redesigned to represent peptide/protein abundance information for all study variables included in the files for each specific assay. Different label and label-free quantitation methods are supported (*e.g.* iTRAQ, TMT). A protein table represents the different expression values of each protein per sample and assay (Fig. 3*D*). In addition, the peptide table shows the quantification values at the peptide level. Users can then select studies or abundance variables as quantification values (Supplemental Fig. 9). The sample information related to each assay (*e.g.* reagent, tissue, description) can also be visualized using the “More” option (Supplemental Fig. 10). The “Quantitation view” also provides a panel to compare different expression values at the protein and peptide level using bar chart plots. This feature enables, for instance, the comparison between the expression values of isoforms of the same protein or different peptides of the same sequence (Fig. 3*D*).

##### The “Summary Charts”

The last tab is devoted to the “Charts view,” comprised by a collection of nine charts for assessing the overall properties of the data stored in the corresponding file (as a part of a dataset). It uses the *inspector-quality-chart* library to provide a quick overview of the data at different levels. Each chart is documented thoroughly in the Supplemental information (Supplemental File S1, Section 3). As a new added key functionality, information in the mass spectrum-related charts can now be filtered for identified, unidentified, target, decoy, or all mass spectra (Fig. 3*B*). The new feature can be used to compare the differences for all the properties between the target and decoy identifications. For instance, the “delta chart” (Fig. 3*D*) represents the distribution of the relative frequency of experimental precursor ion mass (*m/z*) minus the theoretical precursor ion mass (*m/z*). In addition, existing PRIDE “global” data are used to perform quality assessment. For example, the “precursor mass chart” uses the overall PRIDE distribution of precursor masses as a reference (Supplemental File S1, Section 3.6). The newly added chart for quantification results represents the peptide distribution *versus* the study variables available in the file. It then shows the differences between all the replicates and samples for every peptide (Supplemental File S1, Section 3.9). Finally, it is important to note all panels in PRIDE Inspector benefit from comprehensive context-sensitive help modules.

##### Exporting the Processed Results in mzTab Format

mzTab is a lightweight and tab-delimited file format that can contain identification and quantification data. Its goal is to enable the reporting of experimental results, hiding from the scientists (potentially those outside the proteomics field) the most complex details, included in the XML-based files. PRIDE Inspector now provides an option to export the results to an mzTab file (Supplemental Fig. 11). It is important to highlight that this new feature enables the export of original mzIdentML and PRIDE XML data to mzTab, including protein inference information and the correct mapping of protein modifications.

##### Accessing and Searching Data in PRIDE Archive

PRIDE Inspector can be used to access PRIDE Archive datasets directly to either download (available for all datasets) and/or visualize (available for complete PX datasets) public and private datasets (Supplemental Fig. 12). Users can use the functionality “Search PRIDE”. For public data, this is done *via* the recently developed PRIDE Archive web services (47). Importantly, due to the potentially big size of the files that need to be downloaded, the Aspera file-transfer protocol (http://asperasoft.com/) is enabled by default making the speed of file transfers up to 50 times faster than the widely used FTP protocol (which is also supported). This makes the download process much more straightforward, enabling an efficient download of big datasets. In the case of private datasets, journal reviewers and editors can access the files during the manuscript review process by providing a username and password (provided to the submitters after dataset submission).

The new “Search PRIDE Panel” provides similar live search capabilities to the PRIDE Archive website (http://www.ebi.ac.uk/pride/archive/). For instance, it is possible to query by amino acid sequences, protein accession numbers, species and other sample related information, project tags, and protein modifications (including posttranslational modifications) and to split between complete and partial submissions. This new implementation allows querying PRIDE in real time. Compared with the previous version of PRIDE Inspector, users no longer have to wait for new releases of the tool to have access to the latest PRIDE Archive public experiments and projects as they become publicly available (Supplemental Fig. 12).

##### Performance Benchmark, Testing, and Documentation

The performance of an algorithm, library or software component, in terms of time and machine resources (Central Processing Unit, memory, disk), is a crucial aspect on software development (48). We performed a full performance study of the PRIDE Inspector Toolsuite in two different computer settings, using a subset of public PRIDE complete submissions. All the details are included in Supplemental File S1, Section 7. For most of the files, PRIDE Inspector tool performed well, with less than 2 min in average needed to load them. In the case of the more complex identification files (mzIdentML and PRIDE XML), the loading was in average 3 and 1 min, respectively. We anticipate that some of the needed future developments will be focused in improving the performance of the software, as files keep growing in size.

All the libraries, visualization components, and the PRIDE Inspector tool itself have been organized using GitHub repositories. In addition, every component contains mandatory tests for its compilation and deployment, following software good practices (49). Furthermore, every repository has a complete documentation page organized by topics, containing different examples. Additionally, the PRIDE Inspector Tool documentation (https://github.com/PRIDE-Toolsuite/pride-inspector/wiki) provides a set of online video tutorials.

## DISCUSSION AND CONCLUSIONS

The PRIDE Inspector Toolsuite constitutes a big step forward compared with the original PRIDE Inspector tool (19). Although the complexity and variation of proteomics workflows remains a major challenge, the PRIDE Inspector Toolsuite constitutes a major improvement in enabling a user-friendly, comprehensive capture and reporting of proteomics data based on data standards and a key element in facilitating data validation and quality assessment of the increasing number of public datasets available in ProteomeXchange resources.

Although we have mainly focused on demonstrating the use of PRIDE Inspector as a standalone tool, we emphasize that the algorithms and libraries included in the Toolsuite can be combined and used independently in other proteomics tools and workflows (45, 50). For example, many of the libraries (*ms-data-core-api*, *pride-utilities*, *pride-mod*) are components in the current PRIDE submission pipeline (5).

The primary motivations behind the development of PRIDE Inspector Toolsuite were that the software had to be as user friendly as possible, scalable, and easy to maintain through a modular architecture, and well documented. Going beyond this goal, the framework now supports use cases that were absent from the original tool but were in demand by PRIDE users. The current version of the Toolsuite supports the major PSI standards file formats supported to perform PX complete submissions to PRIDE and MassIVE, the current PX resources for MS/MS data. In the case of PX complete submissions, both resources aim to provide all the results in the originally submitted format (*e.g.* mzIdentML, PRIDE XML) and additionally in mzTab. It is envisioned that handling of quantitative information, which has been historically one of the main limitations of proteomics repositories, will happen through mzTab. Mascot's (already available from version 2.5) and future MaxQuant's export functionality to mzTab enables researchers for the first time to routinely include peptide and protein quantification results in their data submissions. By supporting mzTab files, PRIDE Inspector Toolsuite is the first visualization and quality assessment tool for quantitation data based on standard file formats. Although MS-based metabolomics information (both identification and quantification) can already be provided in mzTab files (version 1.0), at present the format is being extended to support this functionality in a better way in the context of the COSMOS (for metabolomics data) (51) and MIRAGE (for glycomics data) projects (52). Therefore, it seems feasible that the PRIDE Inspector tool can be extended in the future to support the visualization of this type of MS data *via* mzTab.

Recently, quality assessment of proteomics results has been discussed extensively (53, 54), including how the final results should be made available in public repositories. The development of the *pride-protein-inference* module (21) as part of the Toolsuite enables the community to analyze the proteomics results (independently of the search engine used). Combined with the fragment ion annotation library and visualization components (Supplemental Fig. 6 and Supplemental File S1, Section 6), these algorithms and visualization components (Supplemental File S1, Section 5) will hopefully enable a better quality assessment at the PSM, peptide, and protein levels.

The modular software architecture, extensive documentation, and free availability of the source code enable any interested third party to add support for an additional data format by simply providing a suitable implementation of the Data Object Model (*ms-data-core-api*). We expect that the new features added, such as possible integration into analysis pipelines, laboratory information management systems software, and the independent integration of new formats, will encourage external groups to develop their own submission pipelines to PRIDE or other PX resources. The widespread use of the Toolsuite ensures its stability, continued development, and community support. It is designed for bioinformaticians and developers with ease-of-use, accessibility, and compatibility in mind, in line with accepted best practices and guidelines in developing bioinformatics software (14, 49).

## Supplementary Material

Supplemental Data

## Footnotes

^1^ The abbreviations used are:

MassIVE: mass spectrometry interactive virtual environment
MCP: *Molecular and Cellular Proteomics*
MS: mass spectrometry
PRIDE: Proteomics Identifications (database)
PSI: Proteomics Standards Initiative
PSM: peptide spectrum match
PX: ProteomeXchange
XML: extensible markup language.
