# Supplementary material for: PRIDE Inspector Toolsuite: Moving Toward a Universal Visualization Tool for Proteomics Data Standard Formats and Quality Assessment of ProteomeXchange Datasets
Source: Mol Cell Proteomics. 2015 Nov 6;15(1):305–17. doi: 10.1074/mcp.O115.050229 (PMC4762524; doi:10.1074/mcp.O115.050229)
Supplement: Supplemental Data [file supp_15_1_305__index.html]

PRIDE Inspector Toolsuite: moving towards a universal visualization tool for proteomics data standard formats and quality assessment of ProteomeXchange datasets — PRIDE Inspector Toolsuite: Moving Toward a Universal Visualization Tool for Proteomics Data Standard Formats and Quality Assessment of ProteomeXchange Datasets — PRIDE Inspector Toolsuite — Supplemental Data 

# PRIDE Inspector Toolsuite: Moving Toward a Universal Visualization Tool for Proteomics Data Standard Formats and Quality Assessment of ProteomeXchange Datasets

## Supplemental Data

- Supplemental File S1 (.pdf, 2.3 MB) - Supplementary Information about the software
- Supplemental Figures (.pdf, 4.3 MB) - Supplemental Figures
