# Supplementary material for: PRIDE Inspector Toolsuite: Moving Toward a Universal Visualization Tool for Proteomics Data Standard Formats and Quality Assessment of ProteomeXchange Datasets
Source: Mol Cell Proteomics. 2015 Nov 6;15(1):305–17. doi: 10.1074/mcp.O115.050229 (PMC4762524; doi:10.1074/mcp.O115.050229)
Supplement: Supplemental Data [file 10.1074_O115.050229_mcp.O115.050229-1.pdf]

Supplementary Information Document Contents

1. PRIDE Inspector Toolsuite Technical Implementation... 1

1.1. General Information 2

2. PRIDE Inspector feature list.. 3

2.1 PRIDE Inspector feature list.. 3

3. PRIDE Inspector charts documentation 10

3.1 Delta m/z... 10

3.2. Peptides per Protein 11

3.3. Number of Missed Tryptic Cleavages 11

3.4. Average MS/MS spectrum 12

3.5. Precursor Ion Charge 13

3.6. Precursor Ion Masses 14

3.7. Peaks per MS/MS spectrum 15

Figure 7: Distribution of the number of peaks per MS/MS spectrum. 16

3.8. Peak Intensity Distribution 16

3.9. Peptide per Ratio 17

4. Protein status documentation.. 18

5. Protein Inference panel.. 20

7. Performance Benchmark 23

8. Abbreviations.. 26

9. References 27

1. PRIDE Inspector Toolsuite Technical Implementation

1.1. General Information

System Requirements:

- Java: JRE 1.7 +
- CPU: 2 gigahertz (GHz) or faster 32-bit or 64-bit processor
- Memory: 4 gigabyte (GB) RAM
- Hard Disk: 1 GB MB available
- Platform: It has been tested in Mac OS X, Linux and Windows

Additional Requirements:

- Internet access is needed to connect to the PRIDE web-services, download private and public PRIDE experiments, and get the protein details from the different protein sequence databases supported.

Project Website:

https://pride-toolsuite.github.io

Utility libraries:

https://pride-utilities.github.io

Online Documentation:

Documentations of all tool and library can be found in the corresponding wiki pages:

- PRIDE Inspector tool: https://github.com/PRIDE-Toolsuite/pride-inspector/wiki
- PRIDE Inspector video tutorials: https://github.com/PRIDE-Toolsuite/pride-inspector/wiki/9.-Video-Tutorials
- ms-data-core-api: https://github.com/PRIDE-Utilities/ms-data-core-api/wiki
- pride-protein-inference: https://github.com/PRIDE-Utilities/pride-protein-inference

2. PRIDE Inspector feature list

The GUI module of the PRIDE Inspector is organised in several views, where each view focuses on a particular aspect of the data. There are currently six views:

- 'Overview' view – shows experimental metadata.
- Protein view – shows protein identifications.
- Peptide view – shows peptides used to generate the protein identifications.
- Spectrum view – shows spectra and chromatograms.
- Quantification view – show quantitative data for both proteins and peptides.
- 'Summary charts' view – provides data chars for assessing data quality.

To enable maximum reusability, each view is implemented as an independent component using Java Swing [http://download.oracle.com/javase/tutorial/uiswing/]. This way, more views can be added easily in the future.

Inspector-mzgraph-browser [https://github.com/PRIDE-Toolsuite/inspector-mzgraph-browser], the component responsible for visualizing spectra and chromatograms, is released and available as a self-contained Java library and distributed as part of the PRIDE Inspector Toolsuite. Both this component and the statistical charts in the 'Summary Charts' view are implemented using the JFreeChart [http://www.jfree.org/jfreechart/] API (Application Programming Interface).

Next to the previous six layers, a generic application management framework maintains the context and information shared by the whole environment. It consists of features frequently required by medium or large Java Swing based applications such as: lifecycle services for background tasks, in-memory caching, event bus, user property management and error handling. The framework is independent from the PRIDE Inspector and therefore can be reused for other rich client applications.

2.1 PRIDE Inspector feature list

The new ones are highlighted in bold letters.

A. Key Features

1. Rapid loading of Peaks files:
    a. mzML

   b. **mgf**
   c. **pkl**
   d. **ms2**
   e. **dta**
   f. **apl**
   g. **mzData**
   h. **mzXML**

2. Rapid loading of identification and quantitation files:
   a. PRIDE XML
   b. **mzIdentML**
   c. **mzTab**
3. **Search, access and download PRIDE Archive 'complete' public experiments through web-services, with search capabilities for modifications, species, disease, tissue, etc**
4. Distinct views on mass spectra, chromatograms, proteins, **peptide-spectrum-matches**, peptides, **protein-groups**, **quantification** and **experiment details**
5. Visualize all spectra and chromatograms with **their fragment annotations**
6. **Automatic annotation of Fragment Ion information**
7. **Visualization of Quantitation Results by supporting study variables and abundances for Quantitation Experiments at protein and peptide levels**
8. Download additional protein details, such as the protein name and the most up-to-date protein sequence (for the following protein sequence databases: UniProt, UniParc, Ensembl)
9. **Visualize original information from search engines, scores, p-values, thresholds and annotations**

10. **Visualization of protein group information in tree tables from original files and also visualization of Protein Inference information using graph representation**
11. **Protein inference algorithm to those experiments without protein group information**
12. A decoy identification filter is available for both the protein and peptide tabs and also **filtering by PSM scores is supported**
13. Display short summary of key measurements on experiment quality like delta masses, **protein modification annotations**
14. Ability to perform an initial data quality assessment using a statistical view with different charts for four different categories: **all spectra, identified spectra, decoy identifications and target identifications**
15. User-friendly download facility for private PRIDE experiments (suitable for journal reviewers and editors)
16. **Metadata about the peptide/protein identification protocols: software properties, parameters, databases, files used, etc**
17. Rich documentation on usage and features

B. Spectrum and Chromatogram Related Features

1. **Automatic annotation for spectra using MS/MS fragment ions (if ion assignments are not included)**
2. Support for various ion types, including co-eluting and immonium ions and amino acid annotation for different fragment ion series within a spectrum
3. Filtering based on types of fragment ions and amino acid annotations
4. Ability to show/hide mass differences for amino acid annotations
5. Configurable fragment ion mass error tolerance for performing the amino acid annotations
6. **Visualization of Mass Table to see the differences between assigned ions and spectrum peaks**

7. **Visualization of the delta mass at Fragment Ion level**
8. Save a spectrum or chromatogram as an image. Supported formats include SVG, PNG, JPEG, GIF and PDF file.
9. Highlight peaks with *m/z* and intensity values in a spectrum.
10. Export spectra data to mgf
11. **Show mass differences for selected peaks and annotated fragment ions, showing the sequence of the assigned peptide**
12. Automatic suggestion on possible amino acids and charges based on mass differences of the selected peaks
13. Highlight peak with its *m/z* and intensity value
14. **Show categorized metadata for each spectrum or chromatogram and annotated precursor ions**
15. Batch loading for experiments containing large number of spectra
16. Easy adjustment on the display size of spectra and chromatogram
17. Show MS level and precursor details for each spectrum

C. Protein and Peptide Related Features

1. Show all protein identifications, together with the **peptide-spectrum-matches** and the spectra used for identification
2. **Show general details about protein identifications (such as search engine, search database, search parameters, number of PSMs, number of unique peptides) from original data**
3. Automatic correction of poorly formatted protein accessions by using web services to retrieve protein names, protein status and protein sequences from different protein databases (for UniProt, UniParc, Ensembl)
4. **Show Protein sequence information for those files that contains sequences such as mzIdentML and mzTab**

5. Calculate the theoretical *pI* for both protein and peptide identifications
6. **Sow the protein group for those files that contains Protein Group information**
7. Calculate delta *m/z* for each peptide (difference between the theoretical and the experimental *m/z*). Highlighting is also done according to the chosen threshold (4 Da)
8. **Show for each peptide the list of PSM with the corresponding information of protein modifications, scores, etc**
9. Hyperlink all valid protein accessions and protein modifications accessions.
10. **Possibility to filter by PSM ranks**

D. Protein Inference Visualization

1. **Display Protein group information and the relation between protein-peptides-PSMs**
2. **Select different topologies to visualize the protein group information**
3. **Collapse and un-collapse peptide and PSMs information**
4. **Highlight the differences between peptides and PSMs**
5. **Filter the data by PSMs scores**
6. **Compute the number of shared peptides and PSMs by protein**
7. **Highlight the overlapping peptides and the protein groups**

E. Decoy Filter Features

1. Set a decoy filter based on position of the decoy filter string for each protein identification
2. Show only decoy protein and peptide identifications
3. Show only non-decoy protein and peptide identifications
4. Undo decoy filter
5. **Set PSM filter**

F. Experiment Detail Related Features

1. Display experiment details, including sample, protocol and instrument configurations. The overview tab is split in three different views: 'Experiment General', 'Sample and Protocol', and 'Instrument and Processing'
2. Information about the peptide/protein identification protocols: databases, software, search parameters, etc
3. **Possibility to export the peptide/protein identifications to mzTab files**

H. Quantification Related Features

1. **Show the quantification method that has been used and the current study variables and abundances for each variable**
2. **Show a summary of sample study variables and the abundances details for each quantification reagent**
3. **Show quantification ratios for both proteins and peptides based on a selected for all the study variables or for abundances depending of the selection of (abundances or study variables)**
4. **Possibility to multi-select peptide isoforms for the same Protein**
5. **Display bar charts based on quantification ratios**
6. **Map filtered proteins identifications to the Ensembl web Karyotype Viewer**
7. **Save/Print the protein quantification data to a file**

I. Summary Chart Related Features

1. **'Delta m/z' distribution for all peptides, including all spectra, identified, non-identified, target and decoy identifications**
2. Histogram on number of peptides per protein identification (percentage of controversial identifications)
3. **Histogram on the distribution of missed tryptic cleavages for all peptides including all spectra, identified, non-identified, target and decoy identifications**
4. Average MS/MS spectrum (for all spectra, or filtering between identified and unidentified spectra)

5. **Histogram on the distribution of precursor ion charge for identified spectra including all spectra, identified, non-identified, target and decoy identifications**
6. **Precursor ion masses distribution for all spectra including all spectra, identified, non-identified, target and decoy identifications. It is also possible to visualize a human, mouse and PRIDE reference curves**
7. **Histogram on number of peaks per spectrum including all spectra, identified, non-identified, target and decoy identifications**
8. **Histogram on peak intensity for all spectra (for all, or filtering between identified and unidentified spectra) including all spectra, identified, non-identified, target and decoy identifications**
9. **Histogram on number of peaks per spectrum including all spectra, identified, non-identified, target and decoy identifications**
10. **Distribution of Peptide per Ratio showing all the quantitation study variables and abundances**
11. Save/Print all charts as images
12. Zoom in/out ability for all the charts
13. Easy navigation between charts

J. Other Features

1. Multiple table row selection for export
2. Sorting for all table columns
3. Show/hide table columns
4. Search content within a table
5. Show/hide the side panel
6. Open compressed (gzipped) PRIDE XML and mzML files

3. PRIDE Inspector charts documentation

PRIDE Inspector Toolsuite 'Quality Chart' is a library to provide quality charts for quality assessment of MS/MS proteomics experiments ranging from spectrum and peptide/protein identifications to quantitation results. It provides at present nine charts: Delta m/z, Number of peptides identified per protein, Number of missed tryptic cleavages, Average MS/MS Spectrum, Precursor ion charge distribution, Precursor ion masses distribution, Number of peaks per spectrum, Peak intensity distribution and Quantitation variables distribution per peptides. For all the charts five different categories are used: unidentified spectra, identified spectra, target identifications, decoy identifications, all spectra.

3.1 Delta m/z

This chart represents the distribution of the relative frequency of experimental precursor ion mass (*m/z*) - theoretical precursor ion mass (*m/z*). Mass deltas close to zero reflect more accurate identifications and also that the reporting of the amino acid modifications and charges have been done accurately. This plot can highlight systematic bias if not centered on zero. Other distributions can reflect modifications not being reported properly. Also it is easy to see the different between the target and the decoys identifications.

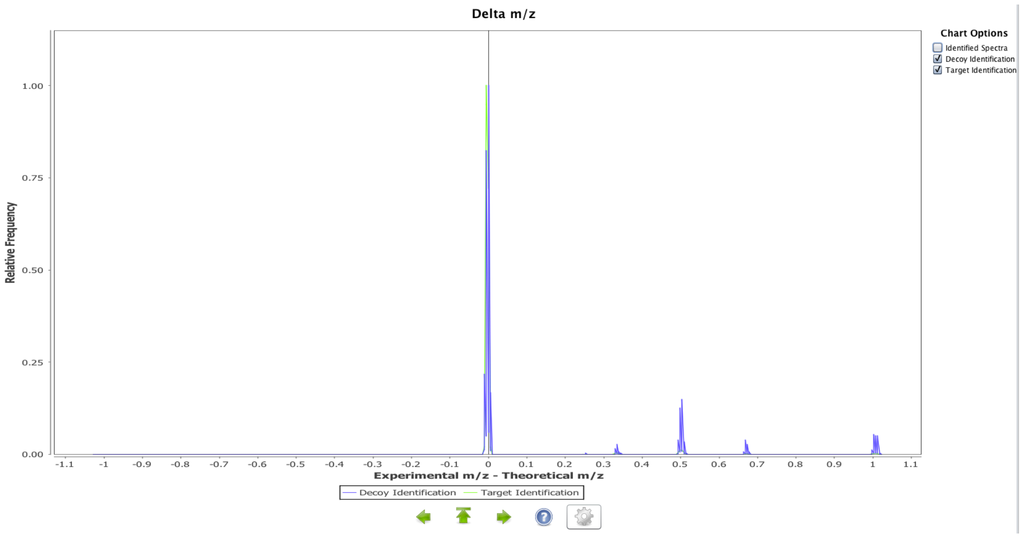

Figure 1: Delta *m/z* distribution: delta mass for target peptides (green) and for decoy peptides (blue).

In Figure 1, we can clearly see that the distribution for this experiment is centred close to zero with for target identifications, but for decoy identifications peaks at 0.5 and around 0.7 m/z units show that are wrong identifications. Peptide sequences, charges and modifications, have been accurately reported and the instrument calibration was fine.

3.2. Peptides per Protein

This is a bar chart displaying the percentage of protein identifications in the whole experiment according to the total number of peptides used to report the identification. Proteins supported by more peptide identifications can constitute more confident results.

Note: To investigate further, in the Protein view, one can sort the proteins by number of peptide identifications.

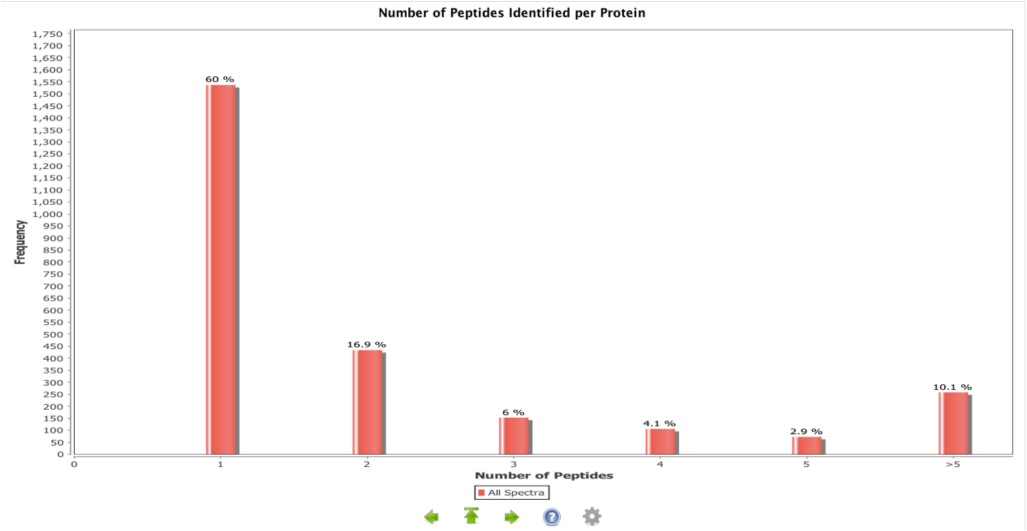

Figure 2: Number of peptides per protein.

In the experiment represented in Figure 2, 60% of the proteins were identified through one PSM only. The rest of the protein identifications, especially the ones with higher peptide numbers can be considered more reliable identifications.

3.3. Number of Missed Tryptic Cleavages

This is a histogram representing the percentage of peptides in the experiment with a different number of missed tryptic cleavages in peptides. This graph is only applicable to experiments where trypsin is used. Two assumptions were made for these calculations: first, the enzyme used in the experiment is trypsin; second, the cleavage rule used by the enzyme is “C-terminal side of K or R except if P is C-term to K or R”. This chart can be used to compare several experiments where the same number of missed cleavages has been used as a parameter for the search, and the same experimental conditions used. Then a dramatic change in the shape of the chart could mean a change in the efficiency of the trypsin used (though many other factors can also be the reason for it, such as a change in the parameters of the search engine, database size and other experimental causes).

In a more practical way, this chart has two immediate applications: first, checking that the search engine is working correctly and the number of missed cleavages found in the identified peptides matches with the "missed cleavages" parameter used in the search engine. Second, by knowing the distribution of this chart, the researcher can adjust the number of missed cleavages used in future searches: e.g. maybe the use of 4 missed cleavages instead of 1 is producing only a 0.1% increase in peptide identifications with searches that are 10 times longer.

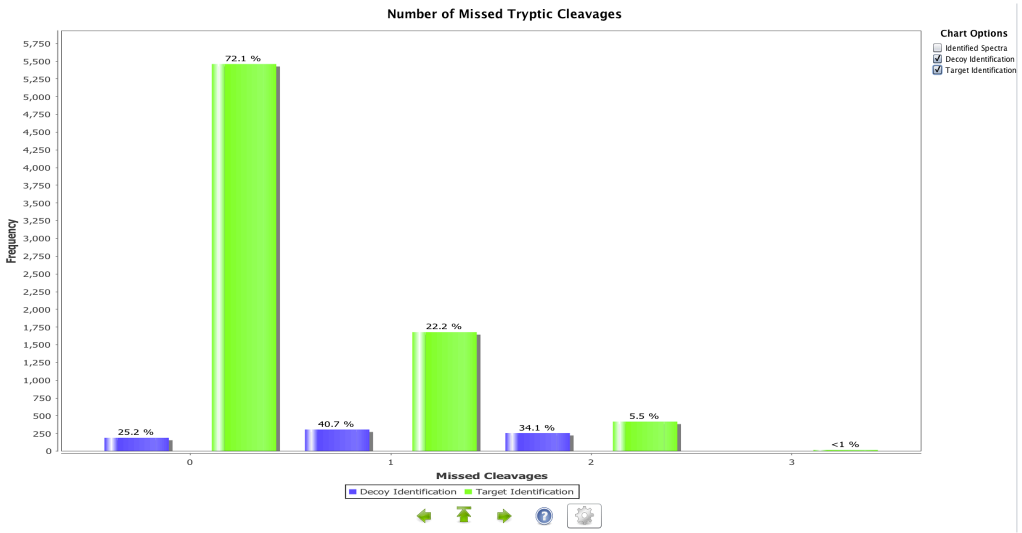

Figure 3: Distribution of peptides containing miscleavages.

Figure 3 shows an example where only about 72% of the target peptides do not have a missed cleavage. However, it is interesting to see that most of the decoy identifications contain missed cleavages.

3.4. Average MS/MS spectrum

This graph is obtained adding all the MS/MS spectra in a given experiment. The result is an averaged spectrum. The highest peaks will reflect abundant and intense peaks in the overall set of MS/MS spectra. Most intense and ubiquitous peaks (both conditions needed) will be displayed here: contaminants, reagents used in the experiment, frequent fragmentations from highly common peptides.

The next chart (Figure 4) shows an example of a public experiment in PRIDE, using iTRAQ reagents for quantification. The zoom has been used to show in detail the highlighted information.

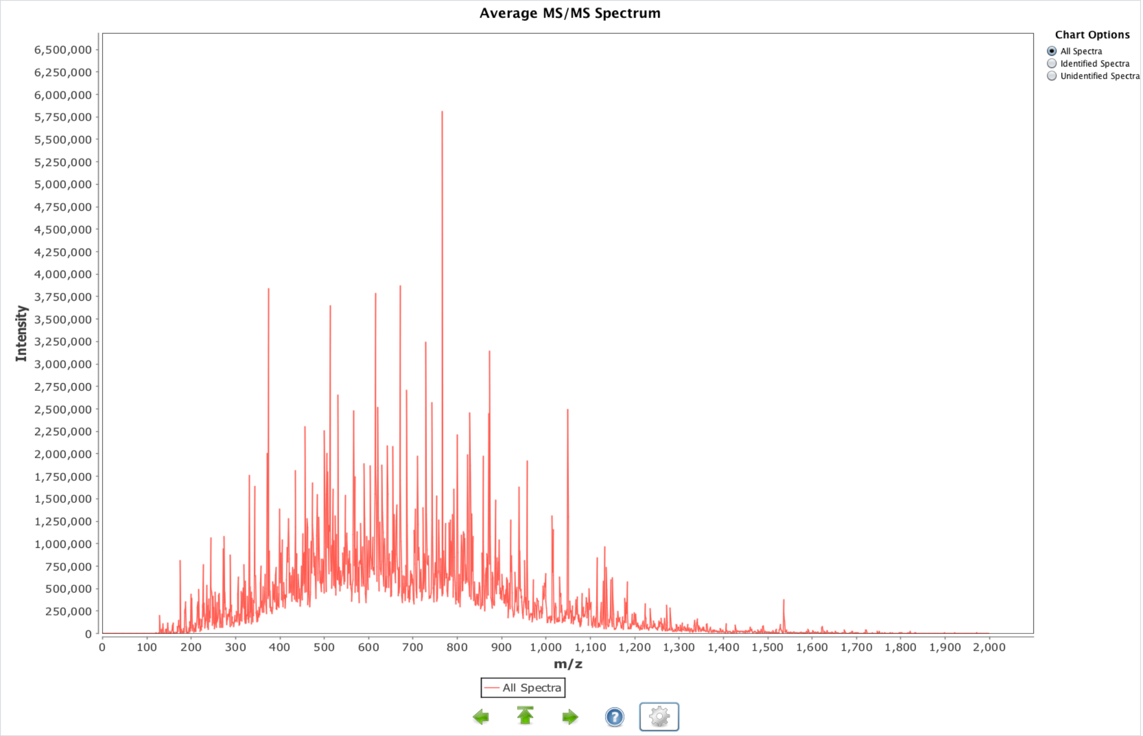

Figure 4: Average MS/MS spectrum considering all the spectra (identified and unidentified).

3.5. Precursor Ion Charge

This is a bar chart representing the distribution of the precursor ion charges for a given whole experiment. This information can be used to identify potential ionization problems including many 1+ charges from an ESI ionization source or an unexpected distribution of charges. MALDI experiments are expected to contain almost exclusively 1+ charged ions. An unexpected charge distribution may furthermore be caused by specific search engine parameter settings such as limiting the search to specific ion charges.

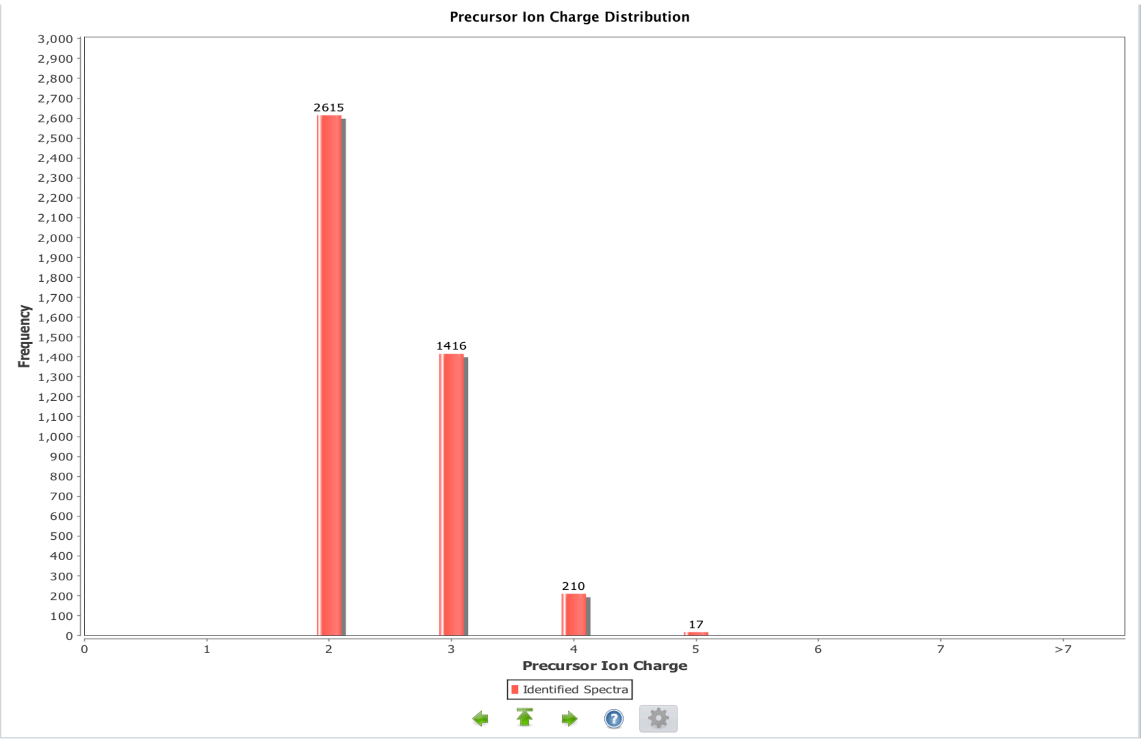

Figure 5: Distribution of Precursor ion charges.

In this ESI experiment there are no single charged ions but only double and triple charged ones.

3.6. Precursor Ion Masses

This chart represents a relative frequency distribution of precursor ion masses for the experiment (red curve) against a reference (if selected by the user). It is possible to filter the information for all, identified and unidentified spectra. Three references are available for the users:

1- Empirically derived precursor ion mass distributions from PRIDE experiments that have a single tryptic digest step annotation associated with them and its upper and lower quartiles. This reference is aimed to provide a species independent distribution.

2- Reference obtained in an analogous way from PRIDE human experiments.

3- Reference obtained in an analogous way from PRIDE mouse experiments.

Experiments that only contained peptides without missed cleavages were ignored as such results are caused by specific search engines parameters and do not reflect the

biological background. These peptides are generally shorter and thus these experiments would shift the overall distribution towards the lower masses.

A curve that lies to the left of the empirical distribution (in a different colour) identifies a disproportionate number of lower mass peptides being identified/ fragmented. In an analogous way, a curve that lies to the right of the empirical distribution identifies a disproportionate number of higher mass peptides being identified/ fragmented. Such alterations may be caused by the general amino acid composition of the organism being investigated, or the digestion protocol used (non-tryptic) but does not necessarily indicate a problem in your experiment.

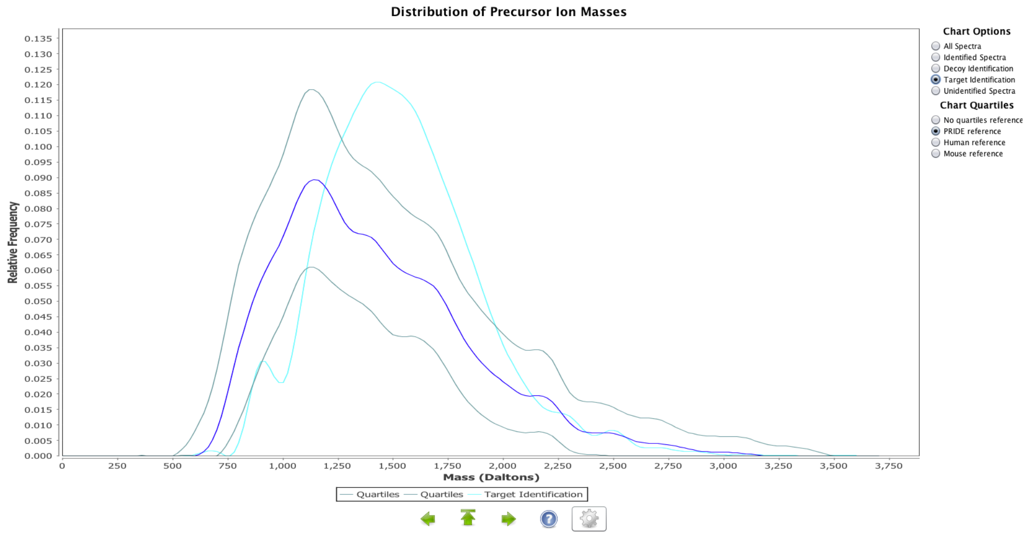

Figure 6: Precursor mass distribution including the PRIDE, human and mouse references.

For human, the average tryptic peptide mass is 1,100 Da. This distribution should encompass this average. A shift to the right in this distribution should be expected due to a number of missed cleavages resulting in higher mass peptides.

3.7. Peaks per MS/MS spectrum

This chart represents a histogram containing the number of peaks per MS/MS spectrum in a given experiment. This chart assumes centroid data. Too few peaks can identify poor fragmentation or a detector fault, as opposed to a large number of peaks representing very noisy spectra. This chart is extensively dependent on the pre-processing steps performed to the spectra (centroiding, deconvolution, peak picking approach, etc). The example shown in Figure 7 shows that poor quality spectra are more likely to be decoy identifications that target identifications.

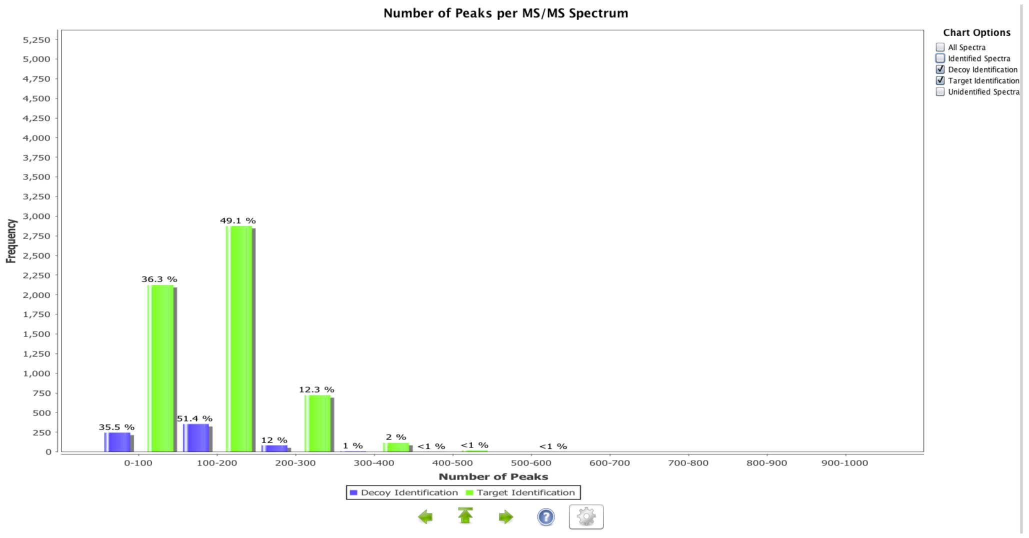

Figure 7: Distribution of the number of peaks per MS/MS spectrum.

3.8. Peak Intensity Distribution

This is a histogram representing the ion intensity vs. the frequency for all MS2 spectra in a whole given experiment (Figure 8). It is possible to filter the information for all, identified and unidentified spectra. This plot can give a general estimation of the noise level of the spectra.

Generally, one should expect to have a high number of low intensity noise peaks with a low number of high intensity signal peaks. A disproportionate number of high signal peaks may indicate heavy spectrum pre-filtering or potential experimental problems.

In the case of data reuse this plot can be useful in identifying the requirement for pre-processing of the spectra prior to any downstream analysis.

The quality of the identifications is not linked to this data as most search engines perform internal spectrum pre-processing before matching the spectra. Thus, the spectra reported are not necessarily pre-processed since the search engine may have applied the pre-processing step internally. This pre-processing is not necessarily reported in the experimental metadata.

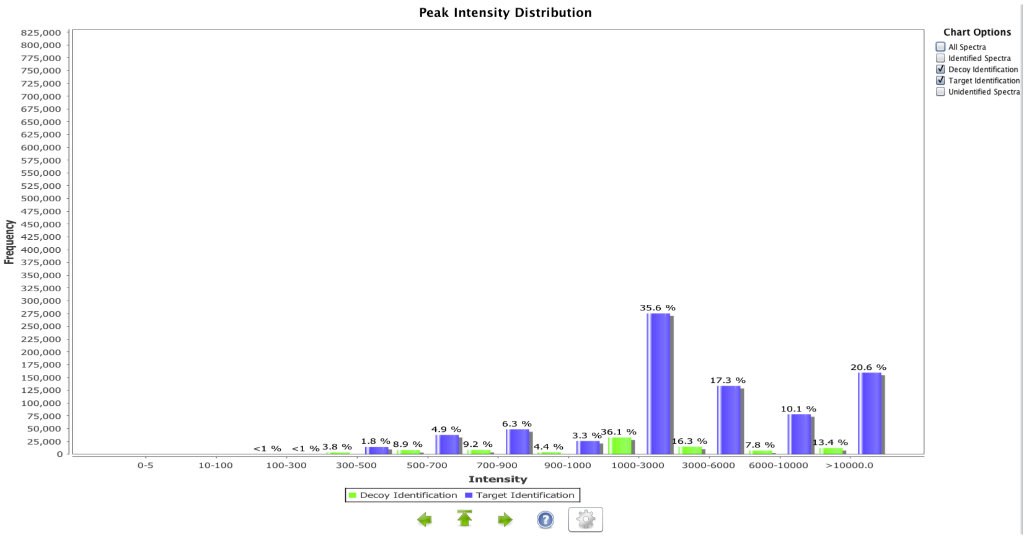

Figure 8: Peak intensity distribution

3.9. Peptide per Ratio

The Peptide per Ratio is a chart representing the peptide distribution versus the study variables in the quantitation experiment. It shows the differences between all the replicates and samples for every peptide. In addition, it shows the relation between different conditions globally. The following example shows the differences between all the samples in an 8-plex iTRAQ experiment.

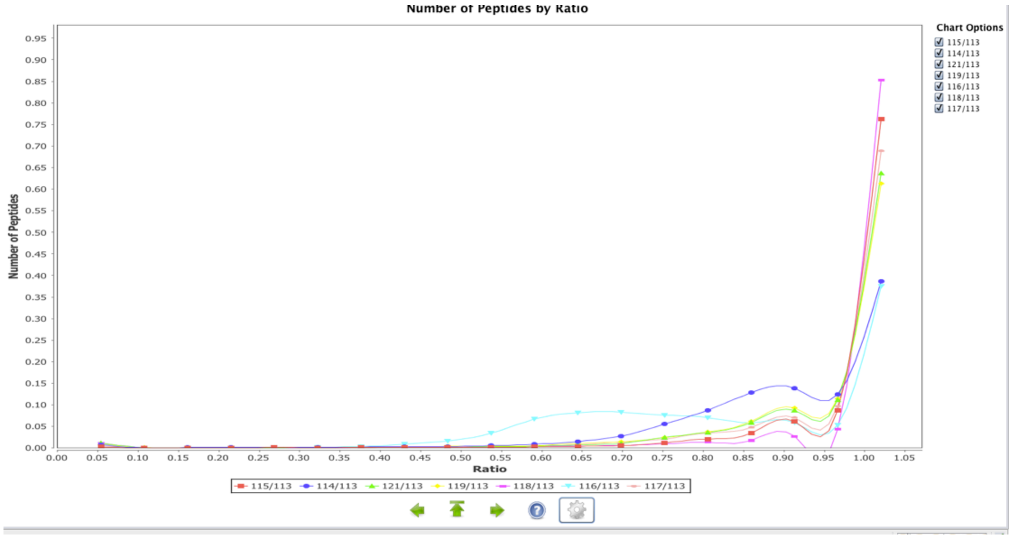

Figure 9: Distribution of Peptide ratios and expression. The number of peptides per ratio for all the samples and variables in the experiment indicates the reproducibility of the experiment and also highlights the differences between conditions.

The PRIDE Inspector Toolsuite charts can be used independently and plugged in Java desktop applications. In addition, it can also be used to generate JSON structures containing the distributions and values of the nine charts. The library has been used in

the PRIDE internal submission pipeline to generate the quality metrics for each submitted file [1].

4. Protein status documentation

In MS proteomics based experiments, potentially identified proteins are reported using the searched database's proprietary identifiers. These identifiers are unstable and can change or may even be deleted over time. The latter happens if, for instance, hypothetical proteins are removed when gene prediction algorithms are updated or new biological evidence is created.

A few years ago we investigated the impact of changing protein identifiers on stored proteomics data over time[9]. We found that in several cases 10-20% of the reported identifiers were no longer valid after only a year after the experimental results had been published. To highlight this problem to the user as well as to keep the reported data usable, PRIDE Inspector Toolsuite has a function to automatically check the reported protein identification's status. To do this we integrated specific components that access the identifications source database and retrieve the current identifier status. If the identifier was only updated, the new accession is automatically displayed in the protein table and the updated sequence retrieved. In some cases, even though a protein's identifier did not change its underlying sequence was altered in the protein sequence database. Therefore, PRIDE Inspector automatically fetches a protein's current sequence and checks whether the reported peptides still fit this identification.

When using the "Obtain Protein Details" feature in the PRIDE Inspector, the status of the protein according to the original database is downloaded in addition to the protein name and protein sequence. It could be one of the following cases:

- **Active**: the protein still exists in the original database, and the details remain unchanged.
- **Unknown**: the protein does not exist in the original database.
- **Deleted**: the protein has been removed from the original database.
- **Merged**: the protein has been merged with other proteins to form a new protein.
- **Demerged**: the protein has been split into two or more proteins.
- **Changed**: there have been some changes on this protein, but the type of the change is unknown.

- **Error**: there is an error associated with this protein.

To summarize, there are three main results for a protein's status: active, changed, and deleted. For UniProtKB (UniProt KnowledgeBase) changed identifiers are subdivided in merged and demerged identifiers. The main reason for the demerging of identifiers is that new identifiers were created for every species a protein was identified in as well as new identifiers for the various genes a protein can come from. The merging of identifiers mainly happens when based on new gene prediction algorithms proteins that were previously believed to be distinct are then considered to actually come from the same gene. The International Protein Index (IPI) database was discontinued in September 2011. Therefore, PRIDE Inspector can only report whether a given identifier was still active in the last IPI release but cannot report on changed or deleted identifiers.

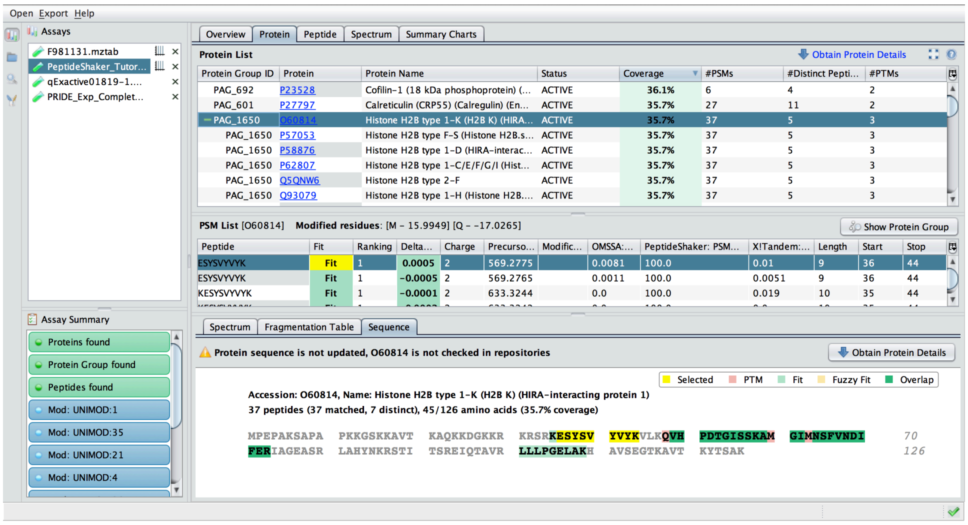

Figure 10: Protein View with Sequence Viewer. The 'Protein View' tab contains three parts: details on the submitted proteins can be observed in the upper section. Information about the corresponding PSM identifications are available in the second section. In the bottom window the 'Sequence Viewer', 'Mass Spectrum Viewer' and 'Fragmentation table' for the selected PSM is displayed.

5. Protein Inference panel

Detailed protein inference information can either be included or not in mzIdentML and mzTab files. In mzIdentML, this information is detailed if 'Protein Groups' are reported. The 'Protein inference panel' shows the identified proteins and the peptides and PSMs included in a particular protein group. The panel can be used to select the proteins and show the identified peptides and PSMs with the corresponding scores and metadata (e.g. modifications, sequence). The 'score threshold panel' enables filtering by PSM score and therefore, to remove evidences below a particular score threshold. The 'Protein inference panel' also enables to see the shared peptides and PSMs between the proteins that belong to a particular protein group.

The visualisation is composed of nodes, representing either proteins/accessions (rectangular, green), peptides (orange, rounded corners), PSMs (light blue, rounded corners) or just connecting nodes (blue circles). The color and thickness of the edges as well as whether a shape is filled and the filling color describes the relation to the selected protein.

A selected protein (or protein group) is highlighted by a red border and is fileld in dark green. All proteins, which have regardingto the set PSM score filter exactly the same PSMs (and thus peptides), are also filled in dark green and have a black border. All peptides belonging to the selected protein are filled in vivid orange, the PSMs in vivid light blue, both also with black borders. Proteins, which are sub-proteins (i.e. all PSMs are also contained in the selected protein) are filled in pastal green without border. Super-proteins (which contain every PSM/peptide of the selected) are also in pastel green with dark green border, and "siblings" (having the same super-proteins) are in pastel green with black border. Peptides and PSMs, which are not contained in the selected but in a super-protein, are filled in pastell with a dark border. Proteins, peptides and PSMs, which have no relation to the currently selected, but are not filtered out, have a thick dark border and no filling. If a PSM, peptide or even protein is filtered out due to the score threshold, the corresponding node has no filling and a thin, black border.

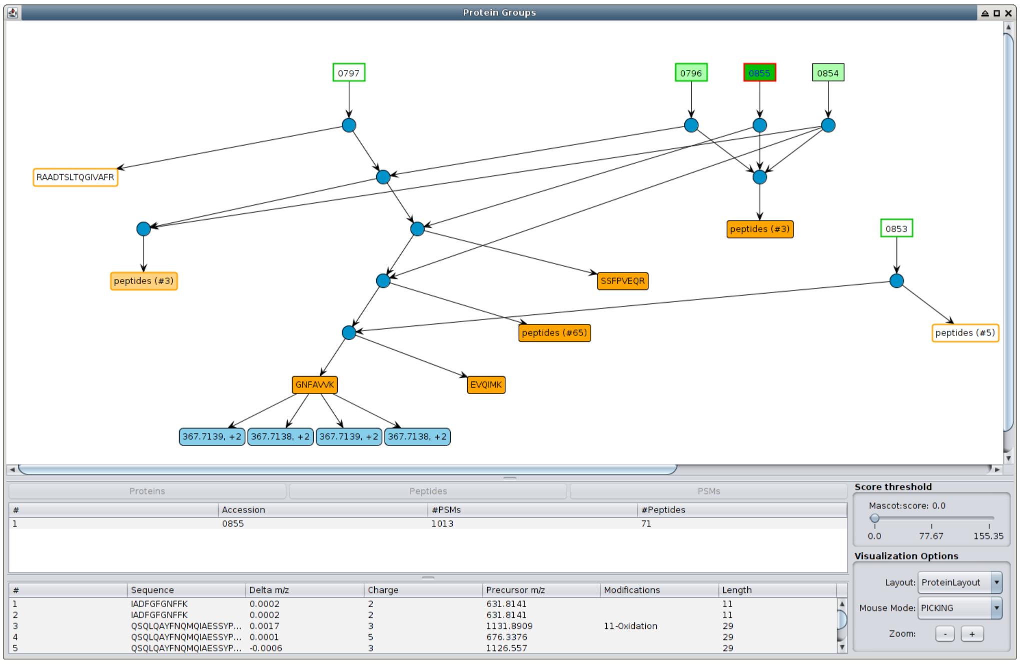

Figure 11: ‘Protein group panel’. When protein inference information is present this panel shows the graph of the protein groups and the final list of reported peptides and proteins.

The ‘protein inference panel’ has options for node picking and transforming and also zoom in and out functionality are enabled. Different colours and shapes are used for proteins (green rectangle), peptides (orange rectangle), PSMs (blue rectangle) and protein groups (blue circles).

6. Fragment annotation algorithm and Fragment table

See the details of the spectrum automatic annotation algorithm in the "Methods" section of the main manuscript. For the matched peaks, a rule-based expert system is used to filter them. These are the main rules used:

1. Ignore annotations for internal fragments, immonium ions and side chain ions;
2. If fragmentation is not coming from HCD or CID instruments, remove the parent ions from the candidate list;
3. If the user chooses water loss or other neutral loss, add annotation with water loss or neutral loss into the candidate list;
4. If peptide charge is 1+, add 1+ annotation charge into candidate list;
5. If peptide charge is 2+, add 1+ and 2+ annotation charge into candidate list;
6. If peptide charge is greater than 2+, Ignore 3+ annotation charge, only add 1+ and 2+ annotation charge into the candidate list;
7. Align different prior weights for different annotation ion types: b ions (100), y ions (99), parent ions (98), a ions (97), x ions (96), c ions (95), z ions (94). Choose the highest weighted annotation, if there is more than one candidate.

The m/z chart shows the delta mass between the annotated ion and the experimental mass of the peak.

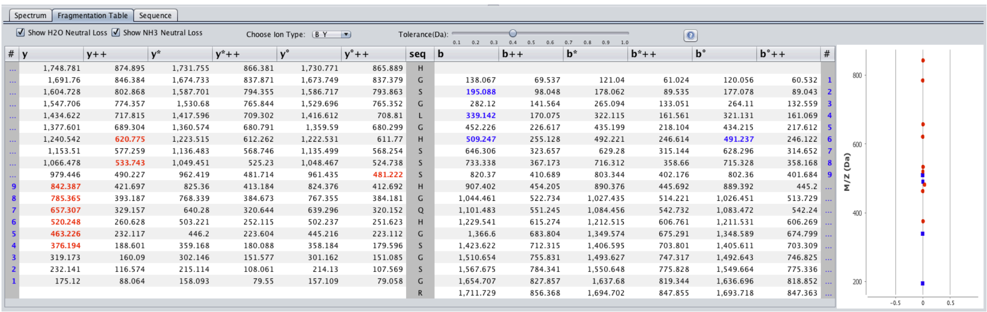

#	y	y++	y*	y*++	y°	y°++	seq	b	b++	b*	b*++	b°	b°++	#
...	1,748.781	874.895	1,731.755	866.381	1,730.771	865.889	H							
...	1,691.76	846.384	1,674.733	837.871	1,673.749	837.379	G	138.067	69.537	121.04	61.024	120.056	60.532	1
...	1,604.728	802.868	1,587.701	794.355	1,586.717	793.863	S	195.088	98.048	178.062	89.535	177.078	89.043	2
...	1,547.706	774.357	1,530.68	765.844	1,529.696	765.352	G	282.12	141.564	265.094	133.051	264.11	132.559	3
...	1,434.622	717.815	1,417.596	709.302	1,416.612	708.81	L	339.142	170.075	322.115	161.561	321.131	161.069	4
...	1,377.601	689.304	1,360.574	680.791	1,359.59	680.299	G	452.226	226.617	435.199	218.104	434.215	217.612	5
...	1,240.542	620.775	1,223.515	612.262	1,222.531	611.77	H	509.247	255.128	492.221	246.614	491.237	246.122	6
...	1,153.51	577.259	1,136.483	568.746	1,135.499	568.254	S	646.306	323.657	629.28	315.144	628.296	314.652	7
...	1,066.478	533.743	1,049.451	525.23	1,048.467	524.738	S	733.338	367.173	716.312	358.66	715.328	358.168	8
...	979.446	490.227	962.419	481.714	961.435	481.222	S	820.37	410.689	803.344	402.176	802.36	401.684	9
9	842.387	421.697	825.36	413.184	824.376	412.692	H	907.402	454.205	890.376	445.692	889.392	445.2	...
8	785.365	393.187	768.339	384.673	767.355	384.181	G	1,044.461	522.734	1,027.435	514.221	1,026.451	513.729	...
7	657.307	329.157	640.28	320.644	639.296	320.152	Q	1,101.483	551.245	1,084.456	542.732	1,083.472	542.24	...
6	520.248	260.628	503.221	252.115	502.237	251.623	H	1,229.541	615.274	1,212.515	606.761	1,211.531	606.269	...
5	463.226	232.117	446.2	223.604	445.216	223.112	G	1,366.6	683.804	1,349.574	675.291	1,348.589	674.799	...
4	376.194	188.601	359.168	180.088	358.184	179.596	S	1,423.622	712.315	1,406.595	703.801	1,405.611	703.309	...
3	319.173	160.09	302.146	151.577	301.162	151.085	G	1,510.654	755.831	1,493.627	747.317	1,492.643	746.825	...
2	232.141	116.574	215.114	108.061	214.13	107.569	S	1,567.675	784.341	1,550.648	775.828	1,549.664	775.336	...
1	175.12	88.064	158.093	79.55	157.109	79.058	G	1,654.707	827.857	1,637.68	819.344	1,636.696	818.852	...
							R	1,711.729	856.368	1,694.702	847.855	1,693.718	847.363	...

Figure 12: 'Fragment ion table' highlighting the assigned ions to the spectra. It also shows each ion delta mass (difference between the experimental and the theoretical mass).

7. Performance Benchmark

In order to know the performance of the PRIDE Inspector tool for different file formats and sizes, we performed a study using public PX "complete" datasets available in PRIDE. First of all, we analysed the size distribution of the main files formats (Figure 13). Most of the files were less than 250 MB in size. For peptide/protein identification files (mzIdentML and PRIDE XML) the average size was less than 200 MB.

In our view, these small sizes are due with different reasons: (i) the MS data files (mzML, mgf) only contain the pre-processed spectra (not the original raw data); (ii) every file contains the identified spectra split by runs, under specific thresholds (e.g. FDR). This is the main reason the size is similar in the case of mgf and mzIdentML; (iii) to avoid issues (e.g. big data sizes), the submitters refined the data before submission (e.g. by removing noise from spectra, or by non providing MS/MS fragmentation annotations). In addition, most of the users did not combine the results of multiple search engines.

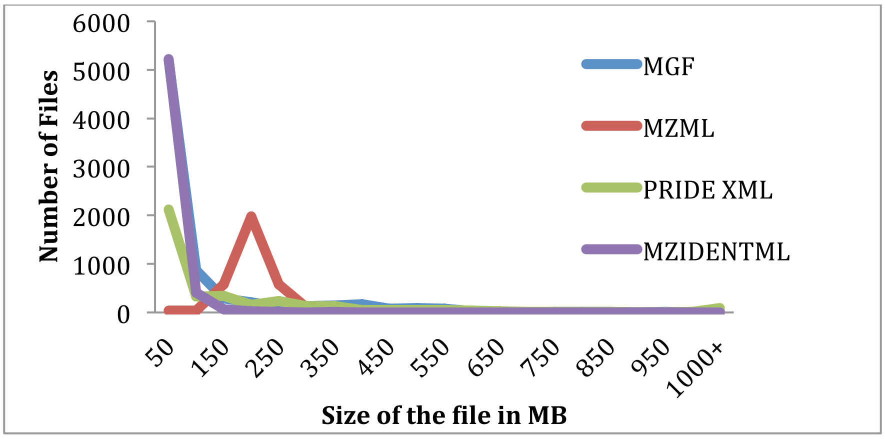

Figure 13: Size distribution of the four main files types included in PRIDE "complete" datasets (mgf, mzML, PRIDE XML and mzIdentML).

Next, we also studied the size distribution of the mzIdentML files with or without protein inference information and MS/MS fragmentation annotation (see Table 1). For instance, we observed that most of the mzIdentML files in PRIDE contained protein inference information (~86%).

File Format/Properties	mzIdentML		mzIdentML	
	Inference	No inference	Fragmentation	No Fragmentation
Total number	4,947	818	35	5,730
Average size (MB)	32	23	103	30

Table 1: Distribution of file sizes and total number of mzIdentML files with/without protein inference and MS/MS fragmentation annotation information in PRIDE.

After studying the file size distribution, we studied the performance of the protein inference algorithms, in the last two versions of the PRIDE Inspector tool (by September 2015). As a result, we found that the version 2.5.1 of the PRIDE Inspector was in average six times faster than the previous version 2.5.0 (Table 2).

	**File size**			
**Software version**	**15 MB**	**30 MB**	**50 MB**	**86 MB**
**version 2.5.0 (min)**	0.82	1.84	9.73	28.12
**version 2.5.1 (min)**	0.221	0.183	2.05	2.16

Table 2: Performance testing of the PRIDE Inspector tool protein inference algorithms, for different file sizes (see https://github.com/PRIDE-Toolsuite/inspector-example-files/tree/master/mzIdentML/noPI).

In addition, we did a full study of the PRIDE Inspector tool performance using files of different sizes, in two different machines and operating systems. The goal was to show the current performance, but also to evaluate the future challenges related to growth in data size and complexity (Table 3).

	**Mac (2.8 GHz I7, 16 GB DDR3, 500 SSD)**				**Dell-Ubuntu (2.4 I5, 8 GB DDR2, HCD 1T)**		
**File type**	File size	First Load	Spectra Load	Chart Generation	First Load	Spectra Load	Chart Generation
**mzML**	500 MB	1.7	3.7	2.5	2.01	2.48	2.67
	300 MB	1	1.7	1.3	1.27	1.58	1.55
	200 MB	0.3	0.7	0.8	0.45	0.63	0.63
	100 MB	0.3	0.7	0.3	0.42	0.40	0.37
	50 MB	0.2	0.3	0.2	0.06	0.24	0.19
		First Load	Spectra Load	Chart Generation	First Load	Spectra Load	Chart Generation
**MGF**	500 MB	0.07	3.7	2.5	0.22	2.20	5.9
	300 MB	0.05	0.6	0.9	0.16	1.51	2.5
	200 MB	0.03	0.4	0.5	0.13	0.95	1.7
	100 MB	0.03	0.2	0.26	0.06	0.58	0.7
	50 MB	0.01	0.1	0.004	0.04	0.36	0.4

		First Load	Peptides Proteins Load	Chart Generation	First Load	Peptides Proteins Load	Chart Generation
**PRIDE XML**	500 MB	0.40	0.12	0.73	0.5	0.16	1.0
	300 MB	0.24	0.2	0.5	0.37	0.2	0.5
	200 MB	0.16	0.1	0.3	0.24	0.1	0.3
	100 MB	0.03	0.02	0.1	0.07	0.06	0.1
	50 MB	0.06	0.03	0.1	0.07	0.06	0.1
		First Load	Peptides Proteins Load	Chart Generation	First Load	Peptides Proteins Load	Chart Generation
**mzIdentML**	300 MB	14.4	1.4	4.2	7.9	0.65	70.4
	200 MB	7.72	0.9	3.1	13.20	2.8	15.4
	100 MB	0.16	0.17	0.10	0.78	0.5	0.4
	50 MB	0.29	1.05	2.71	0.95	0.83	5.3

Table 3: Performance benchmark (measured in minutes) using different file sizes per file type. All the tested files (mzIdentML, PRIDE XML, mgf and mzML) were loaded into the PRIDE Inspector tool using two different machines, as indicated.

The results show that the current PRIDE Inspector tool performs well for most of the files included in the average PRIDE dataset, with less than 1 and 3 minutes in average for loading the spectrum information and for chart generation, respectively. In case of the more complex peptide/protein identification files (mzIdentML and PRIDE XML) the process of loading was in average 3 and 1 min, respectively. It is important to note that the time needed to load an mzIdentML file depends essentially on the internal structure and complexity of the files and in the amount of memory available.

8. Abbreviations

API: Application Programming Interface

CPU: Central Processing Unit

GUI: Graphical User Interface

IPI: International Protein Index

JAXB: Java Architecture for XML Binding

JDBC: Java DataBase Connectivity

JRE: Java Runtime Environment

mgf: Mascot Generic File

PRIDE: PRoteomics IDEntifications (database)

PSI: Proteomics Standards Initiative

PTM: Post-Translational Modification

PX: ProteomeXchange

URL: Uniform Resource Locator

9. References

1. Vizcaino, J.A. et al. The PRoteomics IDEntifications (PRIDE) database and associated tools: status in 2013. *Nucleic acids research* **41**, D1063-1069 (2013).
2. Beausoleil, S.A., Villen, J., Gerber, S.A., Rush, J. & Gygi, S.P. A probability-based approach for high-throughput protein phosphorylation analysis and site localization. *Nature biotechnology* **24**, 1285-1292 (2006).
3. Cote, R.G., Reisinger, F. & Martens, L. jmzML, an open-source Java API for mzML, the PSI standard for MS data. *Proteomics* **10**, 1332-1335 (2010).
4. Griss, J., Reisinger, F., Hermjakob, H. & Vizcaino, J.A. jmzReader: A Java parser library to process and visualize multiple text and XML-based mass spectrometry data formats. *Proteomics* **12**, 795-798 (2012).
5. Reisinger, F. et al. jmzIdentML API: A Java interface to the mzIdentML standard for peptide and protein identification data. *Proteomics* **12**, 790-794 (2012).
6. Xu, Q.W. et al. jmzTab: a java interface to the mzTab data standard. *Proteomics* **14**, 1328-1332 (2014).
