# Supplementary material for: PRIDE Inspector Toolsuite: Moving Toward a Universal Visualization Tool for Proteomics Data Standard Formats and Quality Assessment of ProteomeXchange Datasets
Source: Mol Cell Proteomics. 2015 Nov 6;15(1):305–17. doi: 10.1074/mcp.O115.050229 (PMC4762524; doi:10.1074/mcp.O115.050229)
Supplement: Supplemental Data [file 10.1074_O115.050229_mcp.O115.050229-2.pdf]

Supplementary Figures

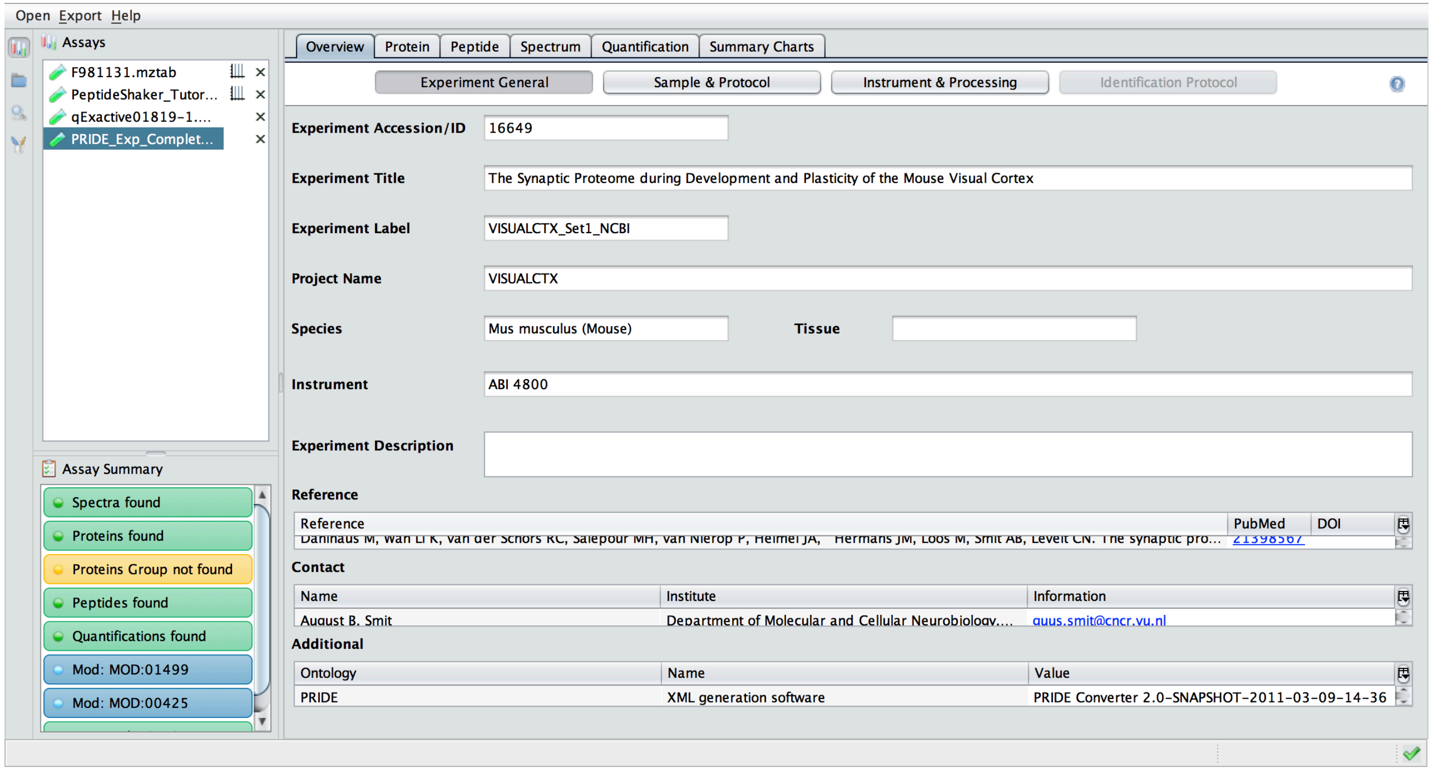

Suppl. Figure 1: PRIDE Inspector 'Overview' panel: 'Experiment General View'. The tab contains basic metadata information about an experimental file: experiment and project titles, contact information, software used for the file generation, and original file format, amongst others.

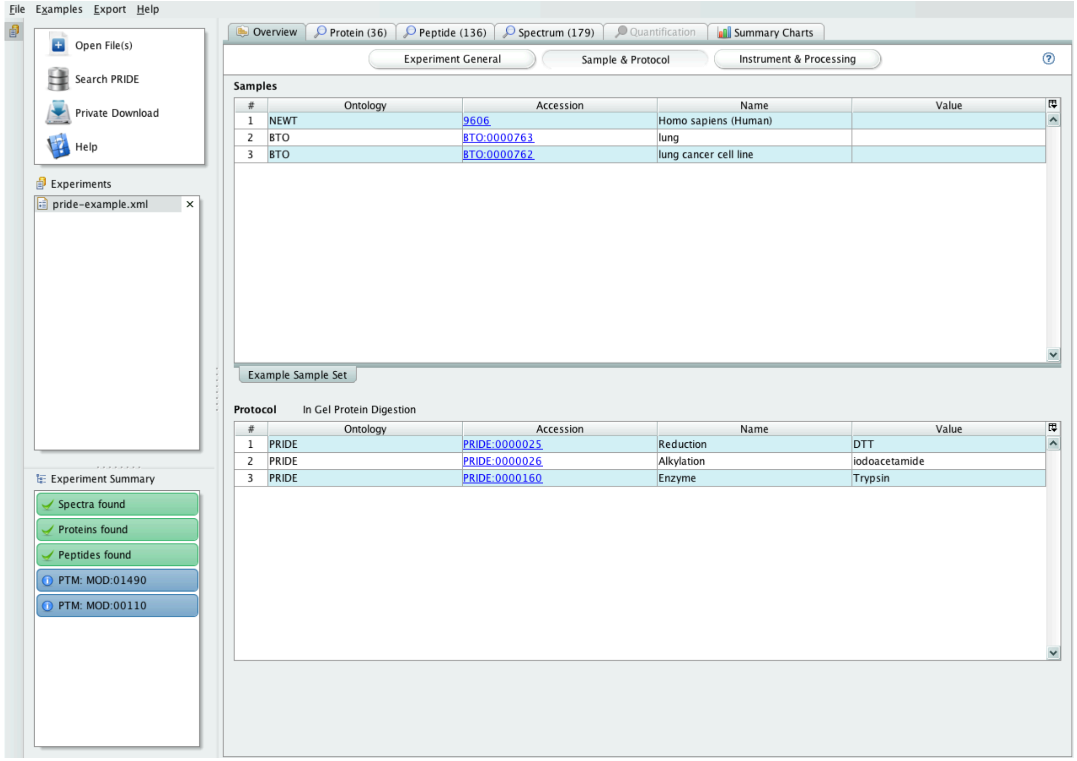

Suppl. Figure 2: PRIDE Inspector 'Overview' panel: 'Sample & Protocol View'. This tab contains metadata information about the sample (species, tissues, etc) and the experimental protocol.

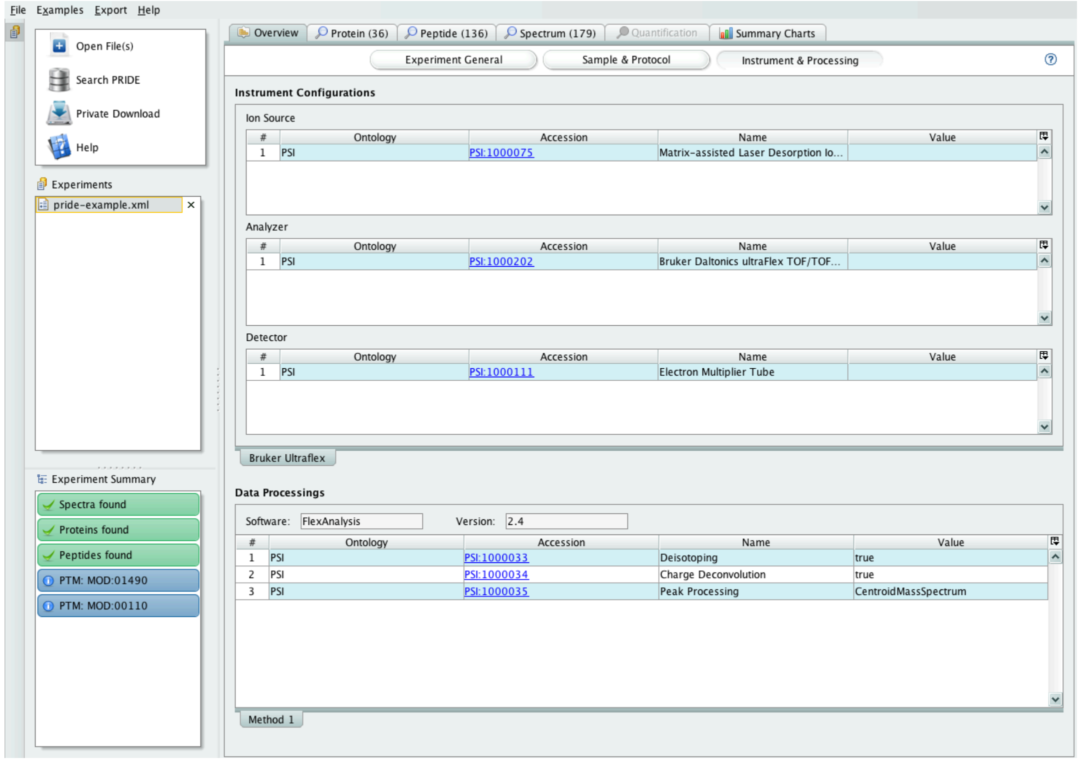

Suppl. Figure 3: PRIDE Inspector ‘Overview’ panel: ‘Instrument & Processing View’. This tab contains metadata information about the instrument configuration and software used.

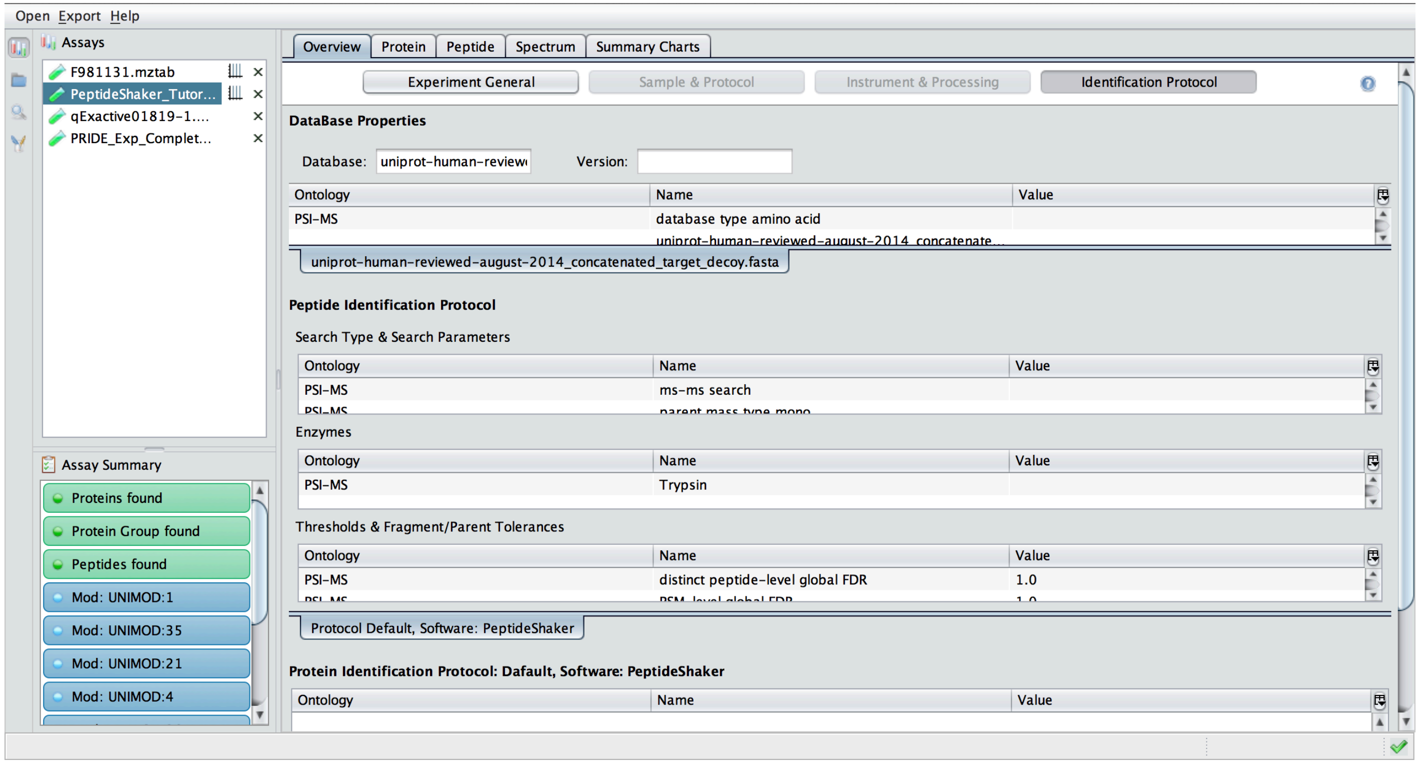

Suppl. Figure 4: PRIDE Inspector 'Overview' panel: 'Identification Protocol View'. This tab contains metadata information about the peptide/protein identification protocols such as search parameters, databases, search engines and software used.

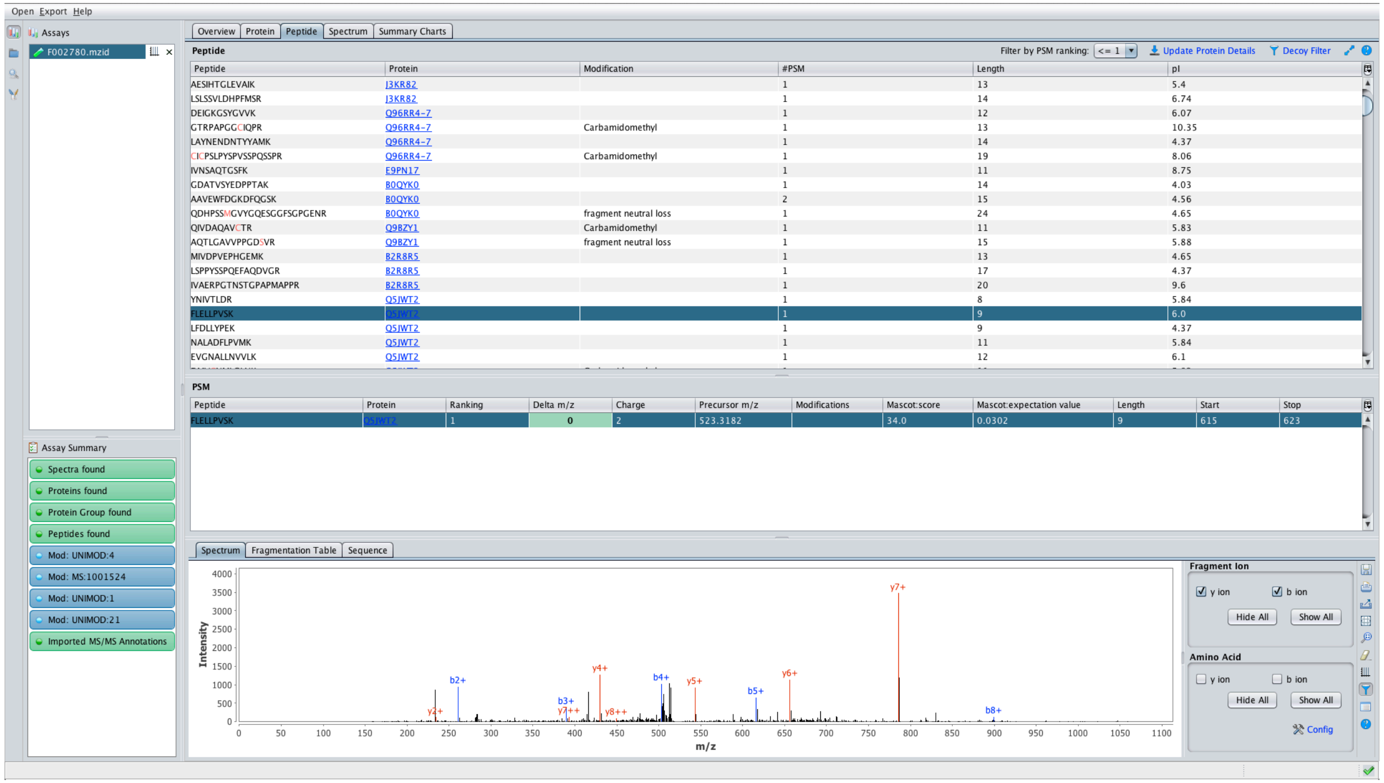

Suppl. Figure 5: 'Peptide View': It shows the peptides identified in the experiment and the corresponding PSMs including different properties such as peptide scores, isoelectric point and sequence length.

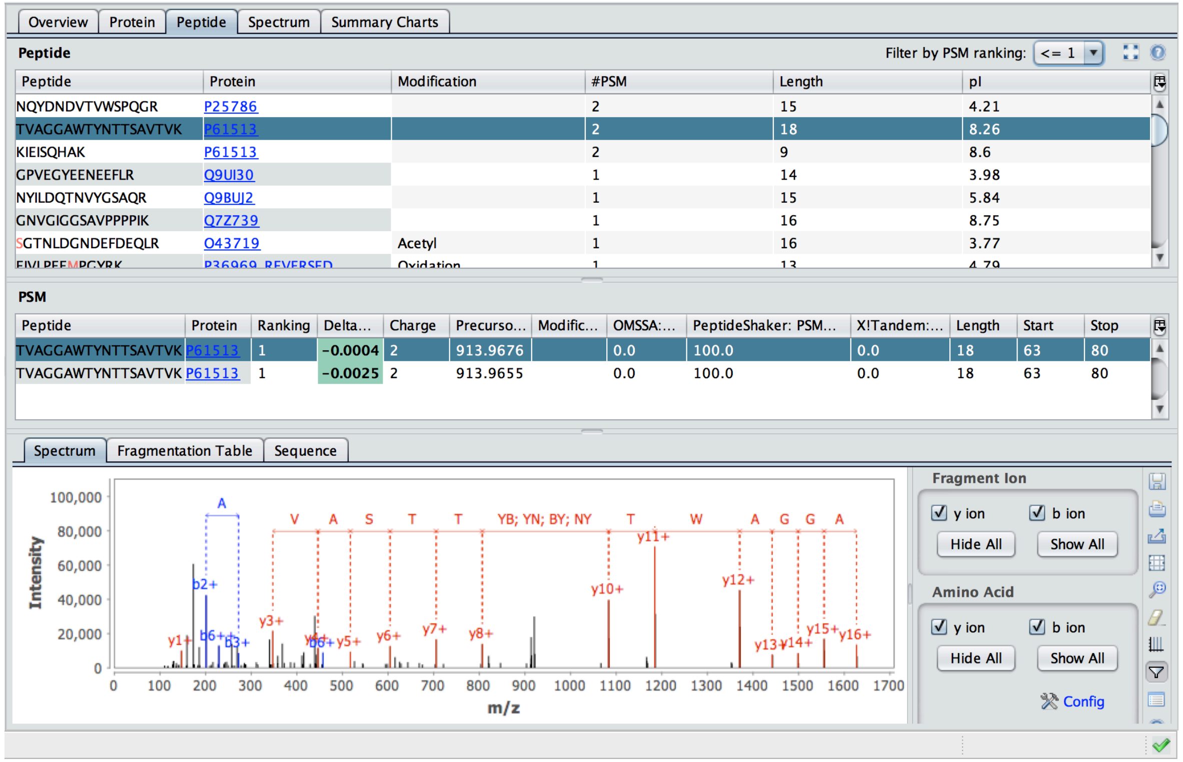

Suppl. Figure 6: The ‘Peptide View’ tab including the mass spectrum fragment annotation viewer: the selected amino acid sequences are highlighted.

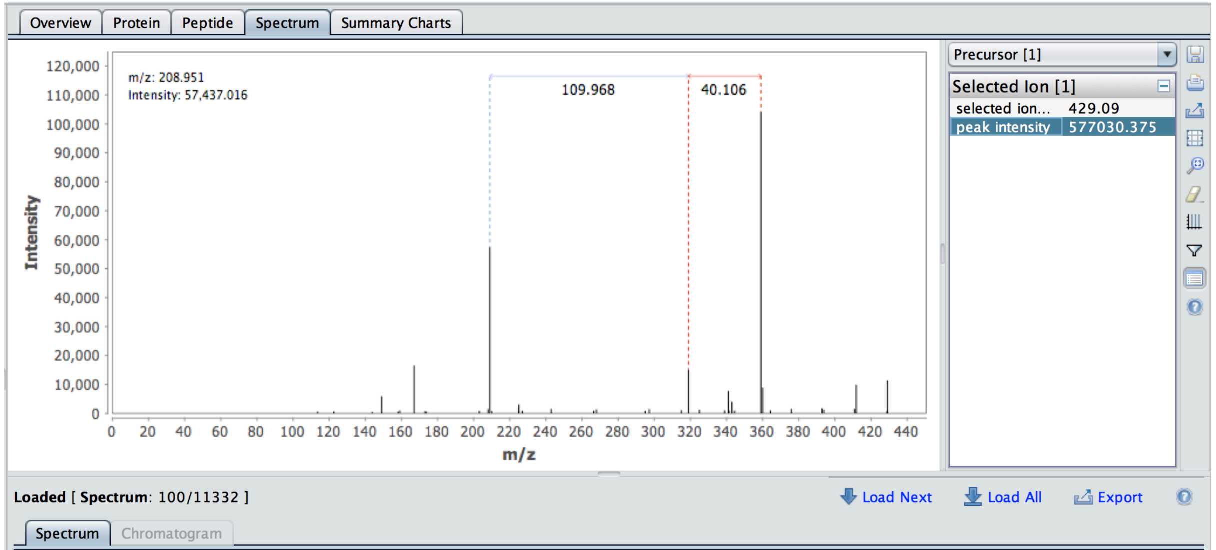

Spectrum ID	MS level	Identified	Precursor Charge	Precursor m/z	Precursor Intensity	Sum of Intensity	# Peaks
1!qExactive018...	2	false		429.09	577030.4	273669.2	39
2!qExactive018...	2	false		327.01	471721.5	490518.9	49
3!qExactive018...	2	false		430.89	484770.3	349615.2	47
4!qExactive018...	2	false		419.32	373636.0	194017.4	35
5!qExactive018...	2	false		462.15	336808.7	657698.6	63
6!qExactive018...	2	false		372.9	325702.8	2422597.0	60
7!qExactive018...	2	false		428.89	577030.4	290777.9	62

Suppl. Figure 7: ‘Mass Spectrum View’. This tab shows all mass spectra (identified + unidentified) included in the experiment. Mass spectrum details and the related scan and precursor information are accessible from the upper right window. In addition it is possible to perform *de novo* sequencing (not shown).

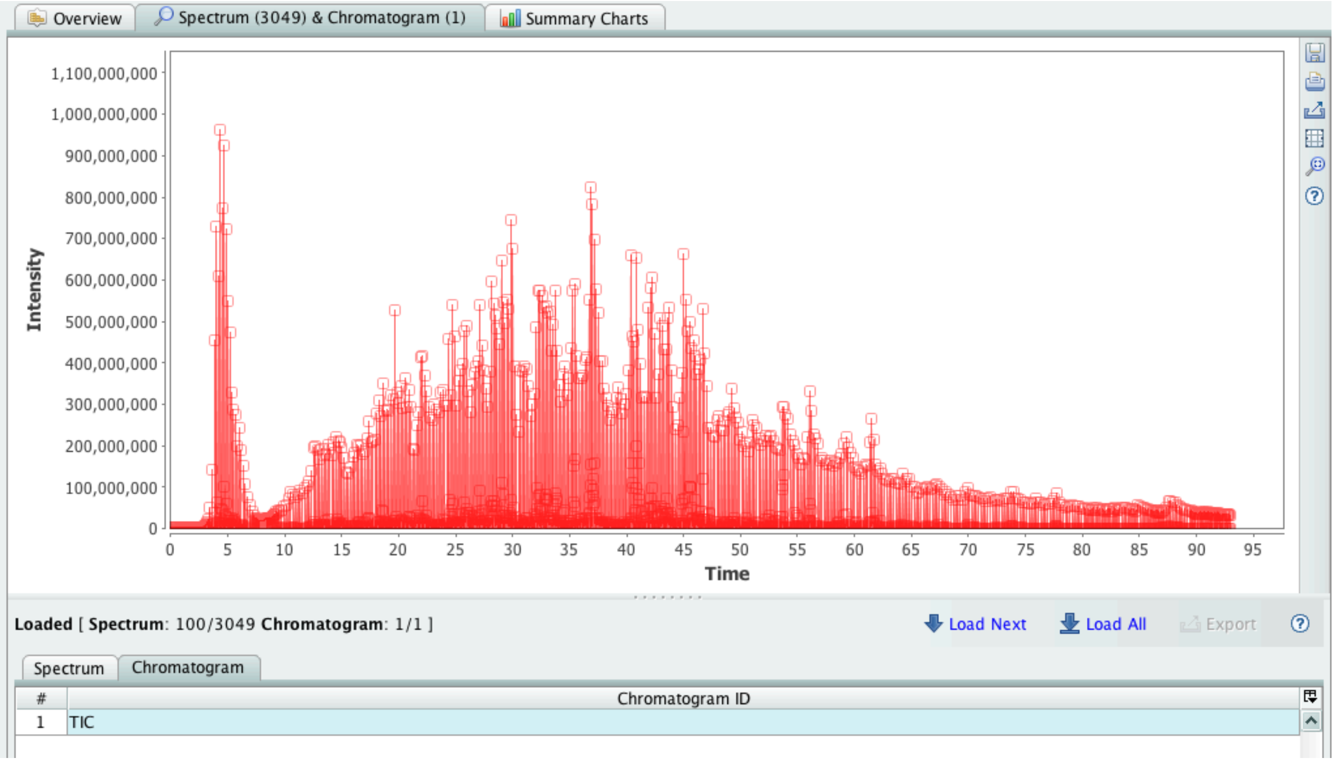

Suppl. Figure 8: ‘ Mass Spectrum View’: If chromatograms are included in the mzML file, PRIDE Inspector can display them in this tab. It must be noticed that chromatograms cannot be included in PRIDE XML and/or mzTab files.

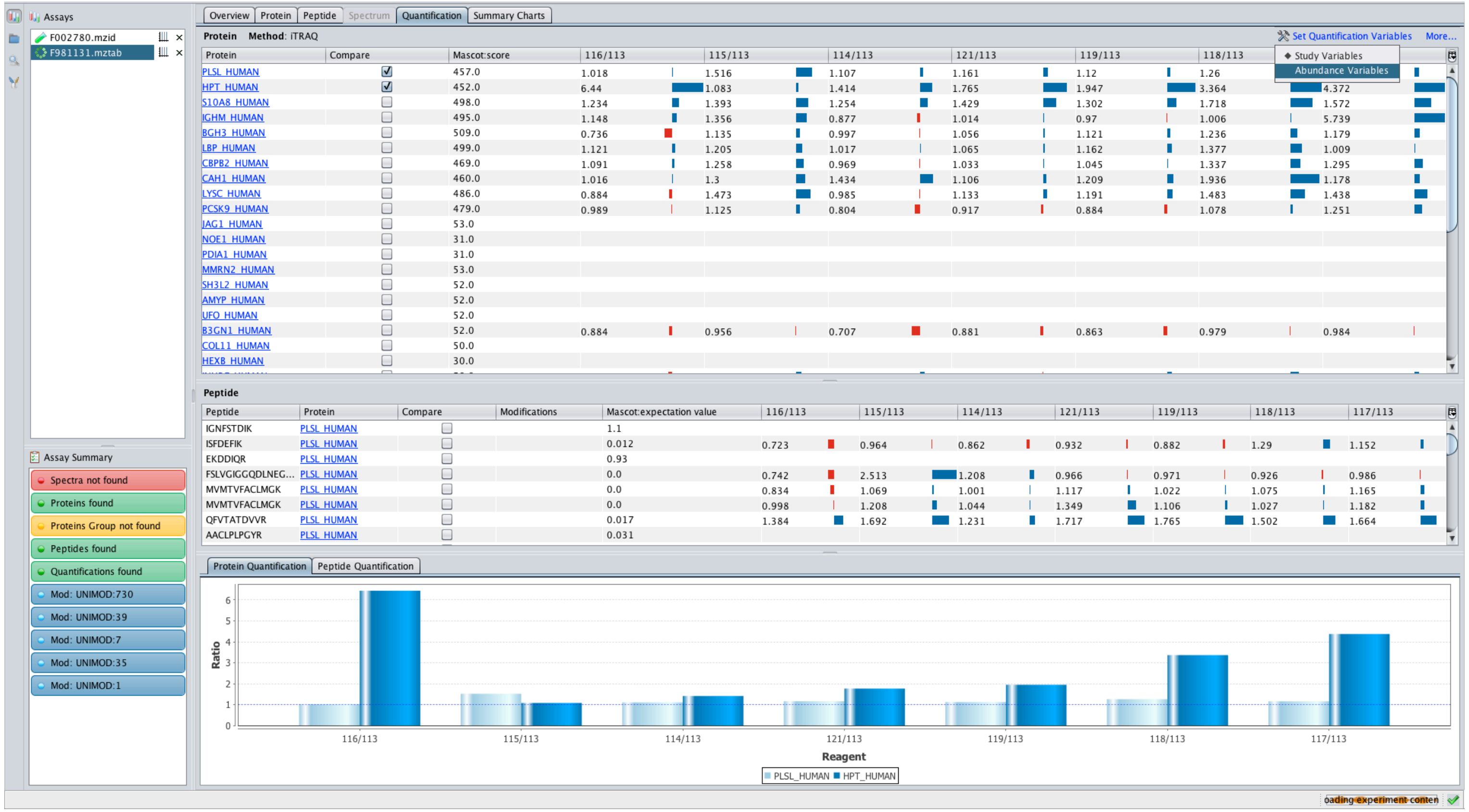

Suppl. Figure 9: “Quantitation View”. This panel shows quantitative information at the protein and peptide level. The “Set quantitation variables” option shows the study variables and abundance variables following the mzTab model.

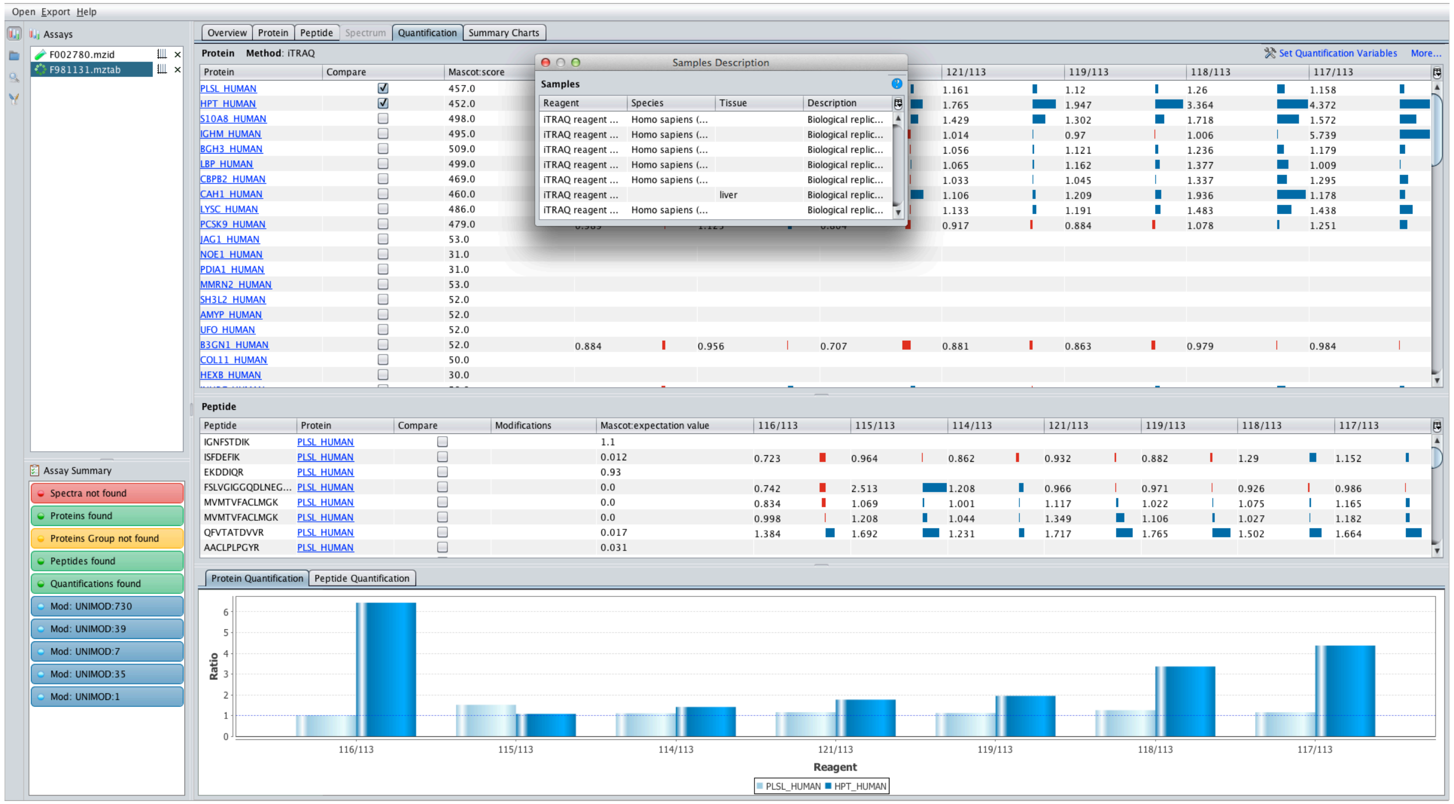

Suppl. Figure 10: "Quantitation Panel". This panel also shows the information about the samples and assays used for quantification purposes (for example, reagents, tissues, and description).

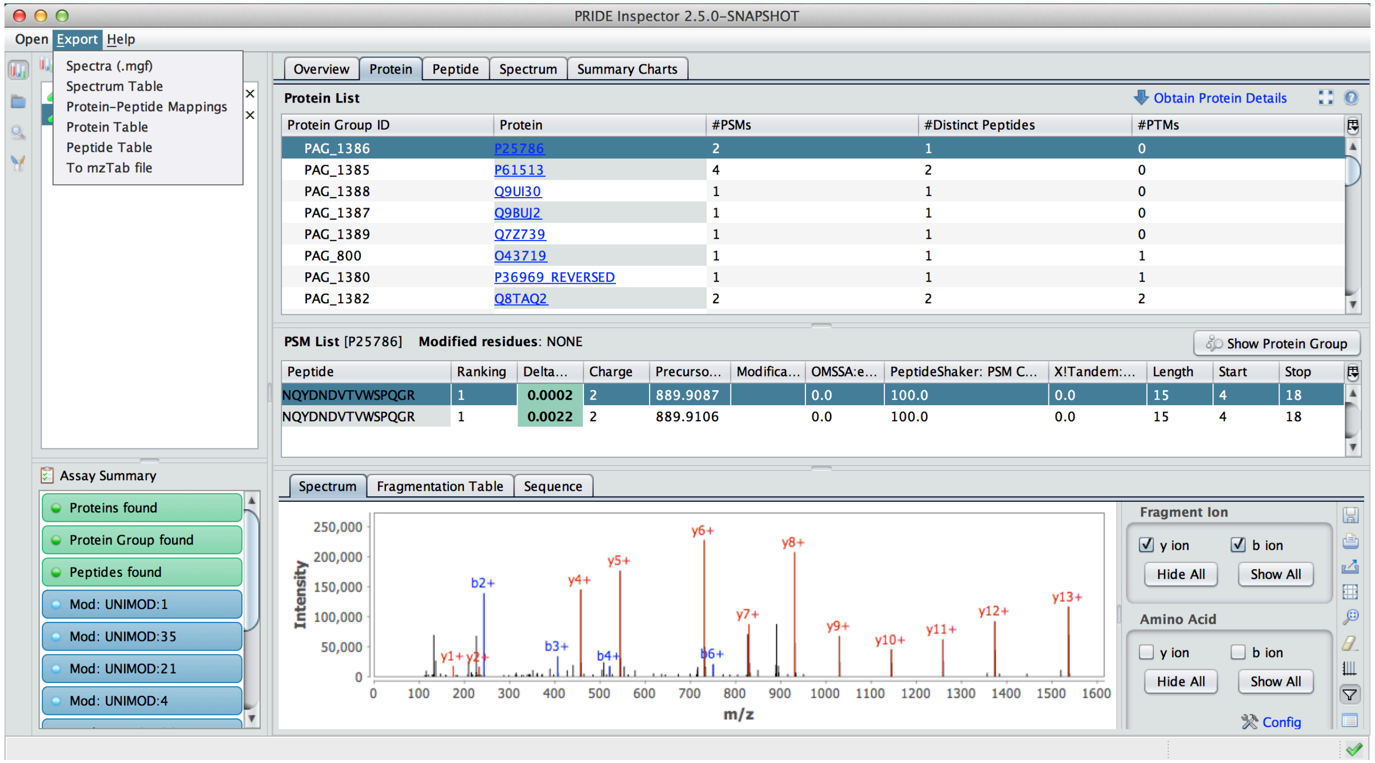

Suppl. Figure 11: PRIDE Inspector tool Export Options. The following export options are available: mass spectra can be exported in Mascot Generic File (mgf) format and as a mass spectrum table. Additionally, the peptide and protein identifications can be exported as 'Protein-Peptide Mappings', and as detailed protein or peptide tables in a tab-delimited format, or in mzTab format.

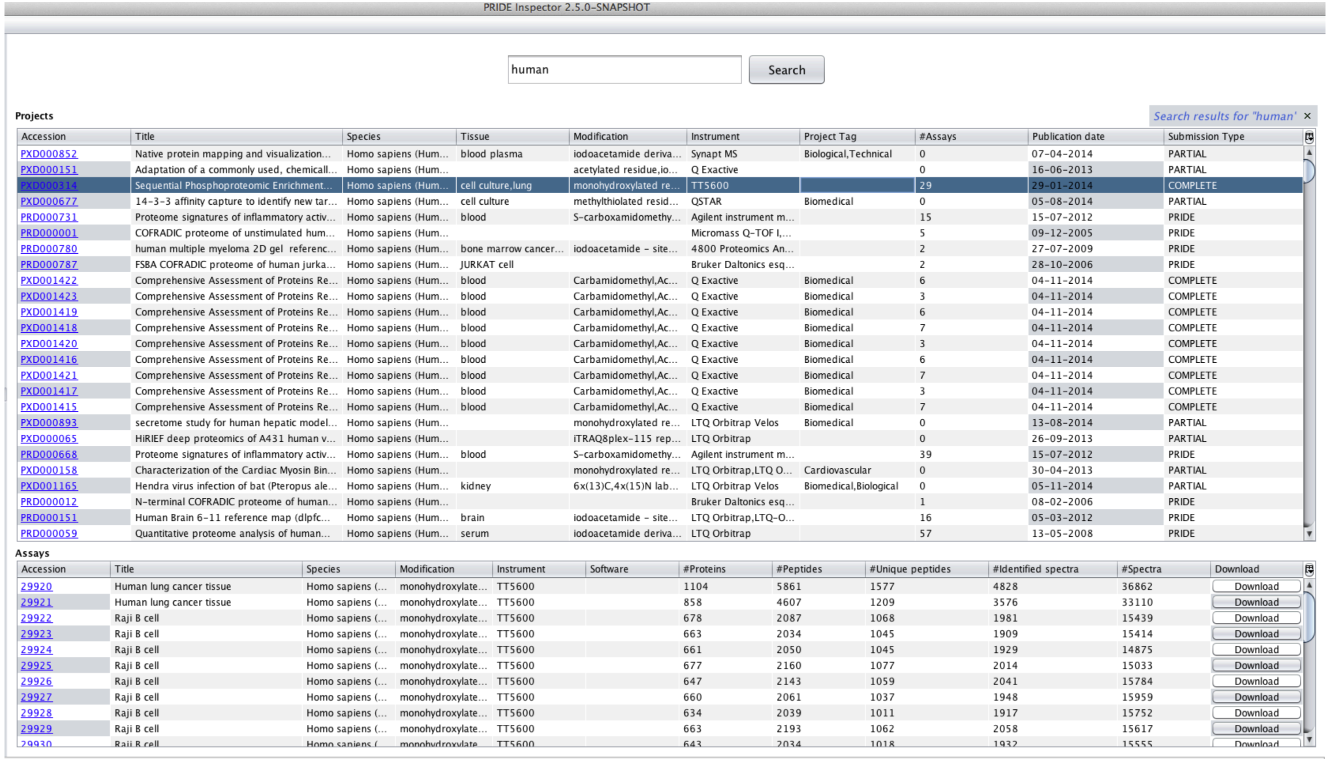

Suppl. Figure 12: PRIDE Inspector 'Search PRIDE' Panel. This panel gives access to the public PX 'Complete' submissions available in PRIDE. Users can search using metadata information such as species, tissue, publication, and project title, among others.
